# Guar bean (*Cyamopsis tetragonoloba*): evaluation as an alternative forage source in ruminants

**DOI:** 10.1007/s11250-026-04983-1

**Published:** 2026-03-12

**Authors:** Arzu Erol Tunç, Engin Ünay, Pınar Özdemir, Gülşen Yıldırım Şenyer, Abdulkadir Erişek, Barış Kılıç, Muhammed İkbal Coşkun, Özgür Güven, Halil Maraş, Yusuf Cufadar

**Affiliations:** 1 Department of Feeds and Animal Nutrition, International Center for Livestock Research and Training, Ankara, Türkiye; 2 Republic of Türkiye Ministry of Agriculture and Forestry General Directorate of Livestock, Ankara, Türkiye; 3 Faculty of Agriculture, Tokat Gaziosmanpaşa University, Tokat, Türkiye; 4 Faculty of Agriculture, Selçuk University, Konya, Türkiye

**Keywords:** Guar bean, Guar hay, Guar silage, In situ, Silage fermentation properties, Ruminant

## Abstract

This study aimed to determine the nutritional values, fermentation characteristics, saponin content, and in situ ruminal digestibility of guar bean (Cyamopsis tetragonoloba) dry matter and various silages adapted to hot climates. Harvesting was performed at the 75-day maturity stage, and the materials were categorized into five groups: guar bean hay (GH), guar bean silage without additives (GS), guar bean silage with 5% molasses (GM), silage containing 2/3 guar bean and 1/3 barley straw (GB), and silage containing 2/3 guar bean, 5% molasses, and 1/3 barley straw (GMB). The silages were fermented in vacuum bags for 60 days. Nutritional composition analyses included dry matter (DM), organic matter (OM), crude protein (CP), fiber fractions (NDF, ADF, ADL), water-soluble carbohydrates (WSC), organic acids (lactic acid, acetic acid, propionic acid, butyric acid, isobutyric acid, isovaleric acid), NH₃-N, CO₂ production, and total saponin levels. In situ incubation trials were conducted in rumen cannulated cows to assess the degradability of DM, OM, and CP. The results showed that GH and GS exhibited high CP levels (15.67–15.84%), low fiber fractions, and high relative feed value (RFV: 320–355), categorizing them as top quality. The addition of straw increased fiber content, subsequently reducing energy and protein content. The inclusion of molasses did not significantly improve fermentation characteristics in this study and had a negative impact on some values. The saponin levels of guar bean products ranged from 3.8% to 4.0% GAE, with high degradability levels observed. The findings suggest that guar dry matter and silage, particularly the GH and additive-free GS groups, offer high nutritional value (RFV: 320–355) and good in situ degradability due to their low fiber content. Consequently, guar bean shows promise as an alternative summer leguminous forage crop in arid conditions. However, considering the presence of high saponin levels, which are potential anti-nutritional factors, further in vivo feeding trials are essential to definitively evaluate its palatability, toxicity, and overall effect on ruminant performance before practical recommendations for feeding systems can be made.

**Graphical Abstract:**

**Figure:**
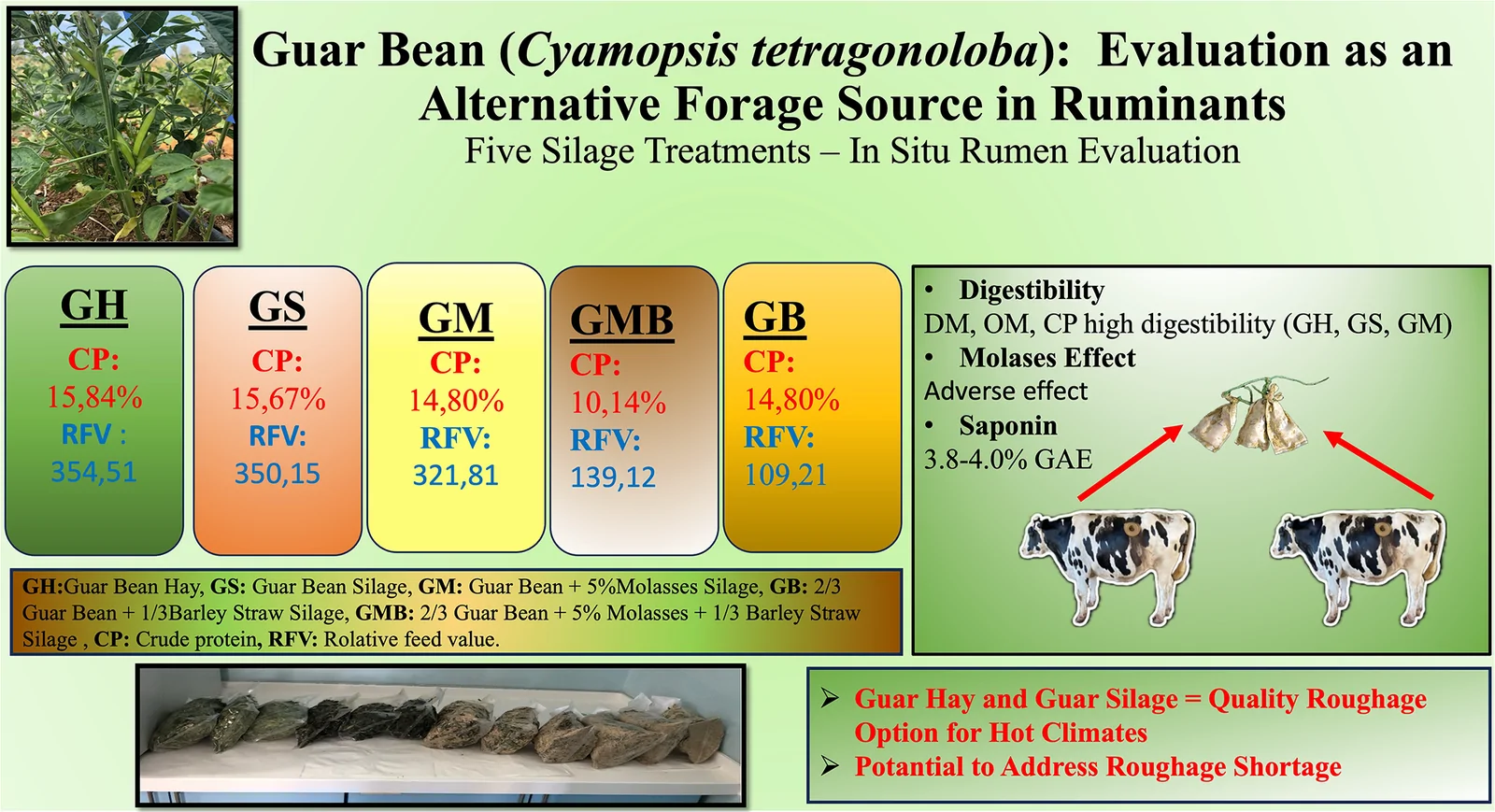

**Supplementary Information:**

The online version contains supplementary material available at https://doi.org/10.1007/s11250-026-04983-1.

## Introduction

Livestock production is increasingly challenged by climate variability, rising feed costs, and concerns over environmental sustainability. The search for alternative protein sources and the implementation of circular resource use have therefore gained growing attention in recent literature. As highlighted by Abbasi (2025), the evaluation of innovative biomass resources plays a central role in meeting global food security and environmental sustainability targets. Ruminant digestive health and productivity are largely dependent on the provision of high-quality roughage. However, in countries where the diversity and availability of roughage sources are limited, there is an urgent need to reassess feeding strategies for ruminants. This underscores the necessity of identifying and investigating alternative forage crops capable of adapting to local ecological conditions, while offering high productivity and nutritional value. Ensuring sustainability in agricultural production, as well as reducing input costs in livestock production, will not only support the introduction of new and high-quality roughage sources but also their widespread adoption, which will critically contribute to the development of modern animal feeding programs.

Among potential candidates, guar bean (*Cyamopsis tetragonoloba)* is a warm season annual legume traditionally grown for both human and animal consumption in South Asia, as well as for the production of valuable guar gum on an industrial scale (Whistler and Hymowitz 1979; Mudgil et al. 2014). Agronomically, it adapts well to hot and dry conditions, requires minimal irrigation, and enhances soil fertility through nitrogen fixation (Rathore et al. 2018). From a nutritional standpoint, the guar plant has the potential to serve as a high-quality summer legume feed owing to its high crude protein (CP) concentration and moderate fiber content, making it suitable for ruminant animals (NRAA 2014). Furthermore, guar can be utilized as green fodder, dry hay, or silage material, thereby providing flexibility in feeding systems, particularly during periods of seasonal feed shortages.

Despite its considerable potential nutritional value, international experimental data regarding the chemical composition and digestibility of guar forage and silage remain limited. When harvested at the appropriate stage of growth, guar plants have been reported to be palatable and rich in nutrients; however, their saponin content, fiber fractions, and other anti‑nutritional factors may adversely affect both intake and digestive efficiency (Mahala et al. 2012; Salama and Nawar 2016). To mitigate these adverse effects and enhance feeding efficiency in ruminants, the use of various additives during the ensiling process has been investigated. Several studies have demonstrated that additives such as molasses (Kehar and Johri 1959) and cereal straw (Reddy and Kumar 2006) can improve the nutritional quality of silage, leading to better animal health and performance. As by‑products, these additives can enhance feed quality at lower cost by utilizing inexpensive and locally available resources, reduce feed expenses, decrease feed intake per unit of production through improved digestibility, and minimize feed losses, thereby indirectly lowering total production costs. Consequently, the use of appropriate silage additives may be considered a strategically important approach in the livestock sector, offering both economic and efficiency benefits.

In line with the goal of reducing the roughage deficit and diversifying summer feed options, this study is designed to evaluate the chemical composition, silage fermentation characteristics, and in situ ruminal degradability of guar bean silages prepared under controlled conditions, both with and without additives. It is anticipated that the findings will contribute to the body of knowledge regarding alternative forage plants and provide practical recommendations for the integration of guar bean into ruminant feeding systems in similar agricultural ecological regions.

## Materials and methods

### Feed material

The feed material used in this study consisted of guar bean (*Cyamopsis tetragonoloba)*, which was cultivated at the International Center for Livestock Research and Training under the Republic of Turkey Ministry of Agriculture and Forestry, General Directorate of Agricultural Research and Policies. The seeds were obtained from a commercial supplier. The guar plants were harvested at 75 days of maturity for evaluation as roughage (see Supplementary Figure S1). From the harvested material, both silage and hay groups were created. The material designated for silage was chopped into pieces measuring 1.5–2 cm and packed into special vacuum-sealed bags for fermentation (see Supplementary Figure S2). Molasses and barley straw were utilized as additives in the silage production process, and the groups were formed according to the type of additive as follows:

- Guar Bean Hay (GH).
- Guar Bean Silage (GS).
- Guar Bean + 5% Molasses Silage (GM).
- 2/3 Guar Bean + 1/3 Barley Straw Silage (GB).
- 2/3 Guar Bean + 5% Molasses + 1/3 Barley Straw Silage (GMB).

### Animal materials

In the study, two Holstein Friesian cows with an average live weight of 600 ± 30 kg, fitted with rumen cannulas, were used at the International Center for Livestock Research and Training. The animals were housed in a tied stall barn, and they were arranged at one-week intervals throughout the experimental period. During the trial, the animals were fed a diet comprising 70% roughage and 30% concentrate, formulated at a maintenance level of x 1.25. They received two daily feedings at 08:00 and 17:00. Fresh and clean water was made available to the animals at all times (Ørskov and McDonald 1979).

### Preparation of silage and hay samples

The feed materials intended for silage (GS, GM, GB, and GMB) were obtained from the same harvest batch and chopped into pieces approximately 1.5–2.0 cm in length in the laboratory. During the grouping process, mixing was conducted manually to ensure a homogeneous mixture for each group. For laboratory-scale ensiling, materials were placed in vacuum-sealable bags (25 × 35 cm), with each bag loaded with approximately 1.0 kg of material according to the experimental treatment to standardize packing density across treatments. Air was removed from each bag using a dedicated vacuum-sealing device (Sales VS100S) and the bags were hermetically sealed to minimize headspace and establish anaerobic conditions necessary for fermentation. Ensiling was initiated based on the measured air-dry matter (Air-DM) contents of the materials; the DM values at the time of ensiling are reported in Table 1.

Twelve silage samples (three subsamples per group for four groups; technical replicates, *n* = 3) were prepared from the homogenized bulk material and allowed to ferment for 60 days. The Guar hay control group (GH) was also obtained from the same harvest batch and manually homogenized. Three replicate samples (*n* = 3) of GH were taken for subsequent analyses.

### Preparation of silage extracts

After a sixty (60) day fermentation period, the silage samples were opened and subsamples were collected for organic acid and pH analyses. The preparation of silage extracts for pH determination and organic acid analysis was performed according to the procedures described by Chen et al. (1994) and Tjardes et al. (2000).

From each silage sample, 50 g of fresh material were weighed and homogenized with 450 mL of distilled water in a laboratory-grade stainless steel blender for 5 min. The resulting mixture was filtered through four layers of cheesecloth to remove solid particles.

### pH measurement

The pH of the filtrate was measured immediately using a glass electrode pH meter (MeterLab, PHM 210).

### Preparation for organic acid analysis and HPLC conditions

For organic acid analysis, 50 mL (or a comparable volume) of the filtered silage extract was acidified with 12 N H₂SO₄ to adjust the pH to the range of 2.0–3.0. Acidification promotes protonation and improves the separation of organic acids. The pH-adjusted extracts were then centrifuged at 26,000 × g (centrifugal force, not rotational speed) for 30 min at + 4 °C (or at room temperature) to obtain the supernatant, which was used directly for HPLC injection.

Analyses were carried out using an Agilent 1100 High-Performance Liquid Chromatography (HPLC) system equipped with an auto-injector and a UV detector. Organic acids were separated on an Aminex HPX-87 H ion-exchange column (300 mm × 7.8 mm i.d.; Bio-Rad Laboratories) under the following isocratic conditions: mobile phase of 0.05 M H₂SOSO₄, flow rate 0.6 mL/min, column temperature 41 °C, injection volume 10 µL, and UV detection at 210 nm, following the method described by Canale et al. (1984). These conditions allowed for the effective separation and quantification of the major organic acids present in the silage extracts.

### Chemical analyses

All feed samples used in the study were dried in an oven at 45 °C for 48 h to determine their air-drymtter weight. The feedstuffs used in the experiment were ground to pass through a 1 mm sieve prior to analysis. Dry matter (DM) (basis 105 °C), organic matter (OM), crude ash (CA), crude protein (CP), and ether extract (EE) contents were determined according to the methods described by AOAC (1995). Air-dry matter, neutral detergent fiber (NDF), acid detergent fiber (ADF), acid detergent lignin (ADL), and crudecellulose (HC) were analyzed following the procedures of Van Soest et al. (1991) using an Ankom 200 Fiber Analyzer.

The water-soluble carbohydrate (WSC) content of the silage samples and guar hay were determined using the phenol-sulfuric acid method as reported by Dubois et al. (1956).

The analysis of ammonia nitrogen (NH_3−_N) was performed utilizing micro-distillation methods on extracts obtained from the silage samples as described by AOAC (1995). The determination of aerobic stability in silages was conducted using the method developed by Ashbell et al. (1991).

To assess the quality of silage and hay, the relative feed value (RFV) index was employed and calculated using the following equations:

- Dry matter digestibility (DMD, %) = 88.9 – (0.779 x % ADF).
- Dry matter intake (DMI, % of body weight) = 120 / (% NDF).
- Relative feed value (RFV) = (DMD x DMI) / 1.29.

Saponin extraction from the feeds was carried out with slight modifications to the method used by Abudayeh et al. (2015). A 2 g sample, ground to pass through a 1 mm sieve, was subjected to extraction with 150 mL of 70% methanol in an ultrasonic bath at 80 °C for 4 h. After extraction, the samples were allowed to settle for 1 h, and the liquid phase was separated, with the extraction repeated twice using fresh methanol. At the end of the process, all methanol phases were combined and evaporated. The residue obtained from evaporation was dissolved in 50 mL of 70% methanol and centrifuged at 10,000 rpm for 10 min. The resulting liquid phase was used for HPLC analysis.

HPLC analysis was conducted according to the method described by Sezgin and Artik (2010), using glycyrrhizic acid as the standard. The HPLC system (HP1100) comprised an Inertsil ODS-P 250 × 4.6 mm 5 μm C18 column and a diode array detector (DAD) operating at 254 nm. The mobile phase consisted of a mixture of methanol, water, and acetic acid in a ratio of 60/34/6 (v/v/v) at an isocratic flow rate of 1 mL/min for a duration of 35 min. Throughout the analysis, the column temperature was maintained at 25 °C. Due to the classification of guar saponins as triterpenoid saponins and the unavailability of their pure standards, the saponin content has been reported as a semi-quantitative value expressed in terms of Glycyrrhizic Acid Equivalent (GAE).

### Rumen degradability characteristics

In this study, the degradability of dry matter (DM), organic matter (OM), and crude protein (CP) of silage and hay samples was determined using the in situ technique described by Ørskov and McDonald (1979).

The experiment was conducted with two rumen-cannulated Holstein cows. All procedures involving animals were conducted in strict accordance with the ethical principles and guidelines set forth by the Local Animal Experimentation Ethics Committee of the International Center for Livestock Research and Training (Decision date and number: 20.12.2017–147). The allocation of feed samples to bags and cows was fully randomized to minimize positional bias inside the rumen.

For the in situ trials, nylon bags (Ankom R510) with dimensions 10 × 20 cm and a pore size of 50 μm were used. Prior to weighing, numbered bags were dried in an oven at 65 °C for 2–4 h until constant weight, cooled in a desiccator, and weighed at room temperature.

Feed samples were ground to pass through a 2.5 mm sieve, and 5 g of each sample was placed into the prepared bags. For each feed type (silage and hay) and each incubation time (0, 2, 4, 8, 16, 24, 48, and 72 h), three technical replicates of each sample were prepared in the laboratory. At the start of incubation, one bag from each replicate was placed in the rumen of each cow, resulting in two independent biological replicates per feed time combination.

Bags were inserted into the rumen in reverse retrieval order to ensure correct exposure times. At the designated incubation period, corresponding bags were retrieved, immediately immersed in cold water to halt microbial activity, then washed under tap water for 5 min to remove rumen residues. After washing, bags were dried in an oven at 65 °C for 48 h, cooled in a desiccator, and weighed again to determine residue weight.

The degradability of DM, OM, and CP for each animal and each incubation period was calculated using the following formula:

$$\begin{aligned}& {\rm{Degradability\:of\:DM}},{\rm{OM}},{\rm{or}}\;{\rm{CP}} \cr&= {{{\rm{Initial\:amount\:of\:DM}},{\rm{OM}},{\rm{or\:CP}}\left( {\rm{g}} \right) - {\rm{Final\:amount\:of\:DM}},{\rm{OM}},{\rm{orCP}}\left( {\rm{g}} \right)} \over {{\rm{Initial\:amount\:of\:DM}},{\rm{OM}},{\rm{or\:CP}}\left( {\rm{g}} \right){\rm{}}}}\times100 \end{aligned}$$

The degradability characteristics of DM, OM, and CP were calculated using the equation proposed by Ørskov and McDonald (1979):

  

$$P=a+b(1-e^-\mathrm{c}\mathrm{t})$$

In this equation, ( P ) represents the degradability of feed DM, OM, or CP at time ( t ), ( a ) indicates the amount of rapidly degradable DM, OM, or CP (washout loss at 0 h), ( b ) is the amount of undegradable but slowly degradable DM, OM, or CP, ( c ) is the degradation rate constant for DM, OM, or CP, and ( t ) denotes time (in hours).

The effective degradability of DM, effective OM, and effective CP were calculated using the NEWAY computer package program (McDonald 1991). The formula for effective degradability (INSE_DM, OM, CP)_ ) is given by:

$$INSE(DM,OM,CP),\%=a+\left(\frac{bc}{c+k}\right)(1-e)^-\left(\mathrm{c}+\mathrm{k}\right)\mathrm{t}$$

In this equation, (k) represents the rate of outflow from the rumen (0.04, 0.06, and 0.08; McDonald et al. 1988).

### Statistical analysis

Data were summarized using descriptive statistics. For variables meeting the necessary assumptions, group comparisons were performed by one‑way analysis of variance (one‑way ANOVA), and significant differences between groups were identified using Tukey’s multiple comparison test. The significance level for all statistical tests was set at α = 0.05. Descriptive statistics were calculated using Minitab (Version 16).

**Table 1:** Nutrient Contents of Guar (*Cyamopsis tetragonoloba*) Hay and Silage Samples

Besin Madde İçerikleri (%DM)	GH	GS	GM	GMB	GB	*P*
DM	93.52 ± 0.12^b^	95.26 ± 0.09^a^	92.86 ± 0.27^b^	94.65 ± 0.13^a^	95.29 ± 0.32^a^	**0.000**
Air-DM	32.45 ± 0.18^b^	35.45 ± 0.18^b^	36.06 ± 0.35^b^	59.13 ± 1.63^a^	62.14 ± 3.20^a^	**0.000**
OM	90.17 ± 0.00^b^	88.77 ± 0.17^bc^	87.83 ± 0.26^c^	91.99 ± 0.26^a^	92.86 ± 0.56^a^	**0.000**
CA	9.83 ± 0.00^b^	11.23 ± 0.17^ab^	12.17 ± 0.26^a^	8.01 ± 0.2560^c^	7.14 ± 0.56^c^	**0.000**
CP	15.84 ± 0.00^a^	15.67 ± 0.39^a^	14.80 ± 0.18^a^	10.14 ± 0.45^b^	8.99 ± 1.11^b^	**0.000**
EE	1.09 ± 0.00	0.86 ± 0.04	0.93 ± 0.14	0.98 ± 0.04	0.82 ± 0.07	0.169
NDF	19.72 ± 0.00^b^	19.92 ± 0.25^b^	21.45 ± 1.55^b^	43.63 ± 0.06^a^	51.86 ± 4.31^a^	**0.000**
ADF	17.64 ± 0.00^c^	17.88 ± 0.32^c^	20.05 ± 1.76^c^	30.35 ± 0.39^b^	37.22 ± 1.92^a^	**0.000**
ADL	3.01 ± 0.00^b^	2.19 ± 0.05^b^	2.71 ± 0.37^b^	3.62 ± 0.20^b^	6.06 ± 0.84^a^	**0.001**
CF	19.61 ± 0.00^b^	17.63 ± 0.32^b^	19.84 ± 1.17^b^	29.39 ± 0.84^a^	32.50 ± 1.43^a^	**0.000**
ME Mcal kg/DM	2.33 ± 0.00^a^	2.37 ± 0.01^a^	2.29 ± 0.04^a^	2.05 ± 0.03^b^	1.96 ± 0.04^b^	**0.000**

^a, b, c^ The differences between the means indicated with different letters in the same row are significant (*P* < 0.05), data are presented as mean ± SEM (*n* = 3 per group), GH: guar hay; GS: guar silage; GM: guar+ molasses silage; GMB: guar+molasses+straw silage; GB: guar+straw silage; DM: dry matter-basis, 105 °C; Air-DM: air-dry matter basis, 65 °C); CA: crude ash; OM: organic matter; CP: crude protein; EE: ether extract; NDF: neutral detergent-insoluble fiber; ADF: acid detergent-insoluble fiber; ADL: acid detergent-insoluble lignin; CF: crude fiber; ME: metabolic energy (Found by calculation; TSE, 1991.)

## Results

### Nutrient contents

The nutrient contents of Guar Hay and Silage samples are presented in Table 1. The study found no significant differences among GH and GS regarding contents of DM, OM, CP, EE, NDF, ADF, ADL, CF and ME. However, the groups with added straw (GMB and GB) exhibited significantly higher levels of DM, OM, NDF, ADF, ADL, and CF compared to the other groups, but they had distinctly lower values for CP and ME (*P* < 0.05). NDF and ADF levels approximately doubled in the straw-supplemented groups. These results indicate that the inclusion of straw increases fiber fractions while decreasing energy and protein content.

### Saponin contents of guar (*Cyamopsis tetragonoloba*) hay and silage samples

The saponin contents of GH and GS were determined to be 3.82 GAE% (38.2 mg/g GAE) and 3.96 GAE% (39.6 mg/g GAE), respectively.

### Dry matter digestibility, dry matter consumption, and relative feed value

**Table 2:** Dry Matter Digestibility, Dry Matter Consumption, and Relative Feed Value of Guar Hay and Silage Groups

GROUPS	DMC (%)	DMD (%)	RFV
GH	6.09 ± 0.00^a^	75.16 ± 0.00^a^	354.51 ± 0.06^a^
GS	6.02 ± 0.08^a^	74.97 ± 0.25^a^	350.15 ± 5.05^a^
GM	5.65 ± 0.39^a^	73.28 ± 1.37^a^	321.81 ± 27.61^a^
GMB	2.75 ± 0.00^b^	65.26 ± 0.31^b^	139.12 ± 0.54^b^
GB	2.34 ± 0.18^b^	59.90 ± 1.50^c^	109.21 ± 10.64^b^
P	**0.000**	**0.000**	**0.000**

^a, b, c^ Differences between means indicated with different letters in the same column are significant (*P* < 0.05), GH: guar hay; GS: guar silage; GM: guar+molasses silage; GMB: guar+molasses+straw silage; GB: guar+straw silage; DMC: dry matter consumption, DMD: dry matter digestibility, RFV: Relative feed value

As shown in Table 2, the groups with added straw (GMB and GB) exhibited significantly lower values of DMC, DMD, and RFV compared to the other groups (*P* < 0.05), indicating that the inclusion of straw reduces the nutritional value of the feed.

### Organic acid levels

**Table 3:** Organic acid levels of silage groups

GROUPS	LA (DM%)	AA (DM%)	PA (DM%)	IBA (DM%)	BA (DM%)	IVA (DM%)
GS	5.58 ± 1.36^a^	2.42 ± 0.05^bc^	0.00 ± 0.00	2.72 ± 0.30^a^	0.54 ± 0.32^ab^	0.10 ± 0.10
GM	1.30 ± 0.49^b^	1.92 ± 0.36^c^	0.07 ± 0.07	0.00 ± 0.00^a^	1.00 ± 0.15^a^	0.19 ± 0.19
GMB	2.67 ± 0.59^ab^	3.36 ± 0.13^ab^	0.00 ± 0.00	1.92 ± 0.20^ab^	0.00 ± 0.00^b^	0.00 ± 0.00
GB	1.02 ± 0.25^b^	4.14 ± 0.20^a^	0.00 ± 0.00	1.45 ± 0.14^b^	0.00 ± 0.00^b^	0.00 ± 0.00
P	**0.013**	**0.000**	0.441	**0.000**	**0.010**	0.564

^a, b, c^ Differences between means indicated with different letters in the same column are significant (*P* < 0.05). data are presented as mean ± SEM (*n* = 3 per group). LA: lactic acid. AA: acetic acid. PA: propionic acid. IBA: isobutyric acid, BA: butyric acid, IVA: isovaleric acid; GS: guar silage; GM: guar+molasses silage; GMB: guar+molasses+straw silage; GB: guar+straw silage

LA content was significantly higher in the GS group than in the GB and GM groups (*P* < 0.05). AA content was significantly higher in the GB group than in the GS and GM groups (*P* < 0.05) and similar to the GMB group. IBA content was significantly lower in the GB group than in the GM and GS groups (*P* < 0.05). BA content was significantly higher in the GM group than in the GMB and GB groups (*P* < 0.05). The differences observed in PA and IVA contents between the treatment groups were not significant (*P* > 0.05) (Table 3).

### Silage fermentation characteristics

**Table 4:** Silage fermentation characteristics of silage groups

Silage Fermentation Characteristics				
GROUPS	pH	CO_2_ (g/kg DM)	NH_3−_*N* (TA%)	WSC (g/kg DM)
GM	8.00 ± 0.05^a^	0.36 ± 0.00^b^	0.25 ± 0.01^a^	7.45 ± 1.10^b^
GMB	5.74 ± 0.23^b^	0.59 ± 0.02^a^	0.10 ± 0.01^b^	48.18 ± 3.56^a^
GB	6.58 ± 0.04^b^	0.62 ± 0.03^a^	0.11 ± 0.02^b^	40.94 ± 10.01^a^
GS	6.09 ± 0.33^b^	0.36 ± 0.00^b^	0.14 ± 0.02^b^	8.64 ± 0.88^b^
P	**0.000**	**0.000**	**0.001**	**0.001**

^a, b^ Differences between means indicated with different letters in the same column are significant (*P* < 0.05), data are presented as mean ± SEM (*n* = 3 per group), GS: guar silage; GM: guar+molasses silage; GMB: guar+molasses+straw silage; GB: guar+straw silage; WSC: water-soluble carbohydrate content

Table 4 presents the fermentation characteristics of the silage. The highest pH value was found in GM, which was statistically different from all other groups. In terms of aerobic stability, the straw-containing groups (GMB, GB) produced significantly more CO₂ compared to GM and GS. The NH₃-N ratio was highest in GM. Additionally, the WSC levels in straw-containing silages were significantly higher compared to the other groups.

### Organic matter, dry matter, and crude protein degradability according to incubation hours

**Fig. 1:**
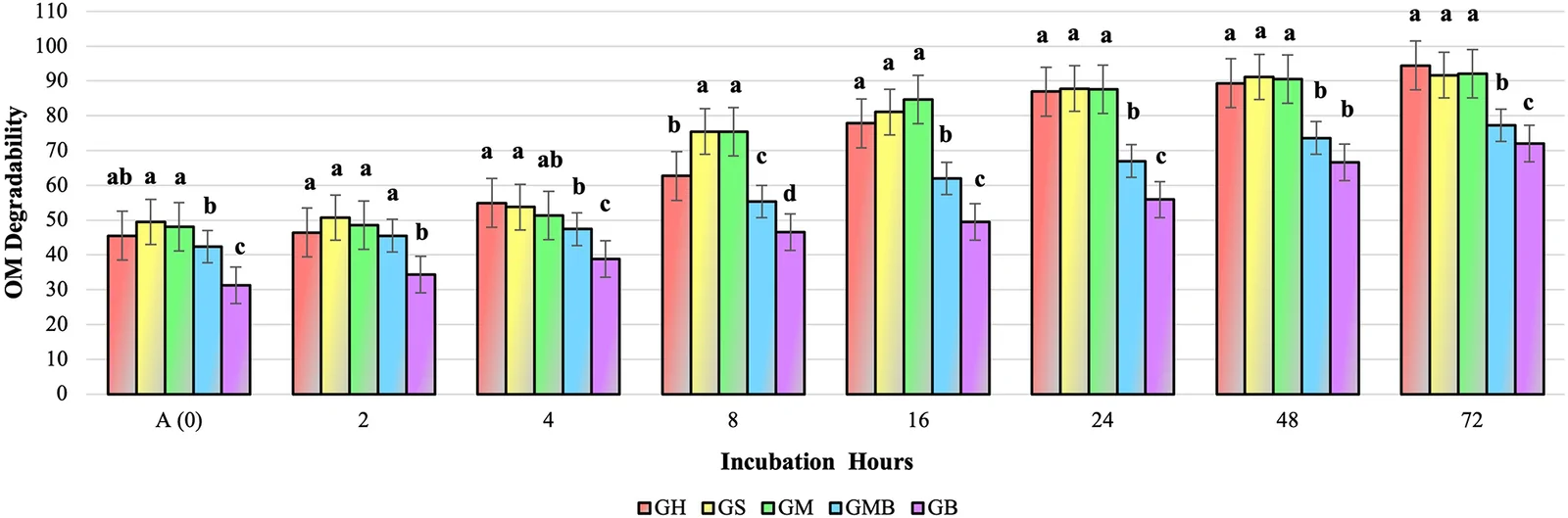
OM degradability of feed samples according to incubation hours (%). ^a, b, c^The difference between values carrying different letters in the same column was found to be statistically significant (P<0.05), n = 2 biological replicates; each value is the mean of three technical replicates per animal, A: Water-soluble fraction of crude protein, washing loss; GH: guar hay; GS: guar silage; GM: guar+ molasses silage; GMB: guar+molasses+straw silage; GB: guar+straw silage

When examining the OM degradability results in relation to incubation hours in Fig. 1, it was observed that the highest wash loss occurred in the GS group, followed closely by the GM group, with no statistically significant difference found between these two groups. The groups containing straw (GMB and GB) demonstrated significantly lower OM degradability compared to the other groups in terms of both wash loss and across all incubation hours, and this difference was found to be statistically significant. The lowest values were detected in the GB group at the 2nd, 4th, and 8th hours, while the GS and GM groups generally reached the highest values. In general, the dry matter degradability values for the GH, GS, and GM groups yielded similar results; however, the differences between these three groups and the straw containing groups were found to be significant. At the seventy second hour, the highest OM degradability was observed in the GH group, while the lowest value was recorded in the GB group. Overall, it can be concluded that the GH, GS, and GM groups exhibit a similar OM degradability profile, whereas the inclusion of straw significantly reduces degradability.

**Fig. 2:**
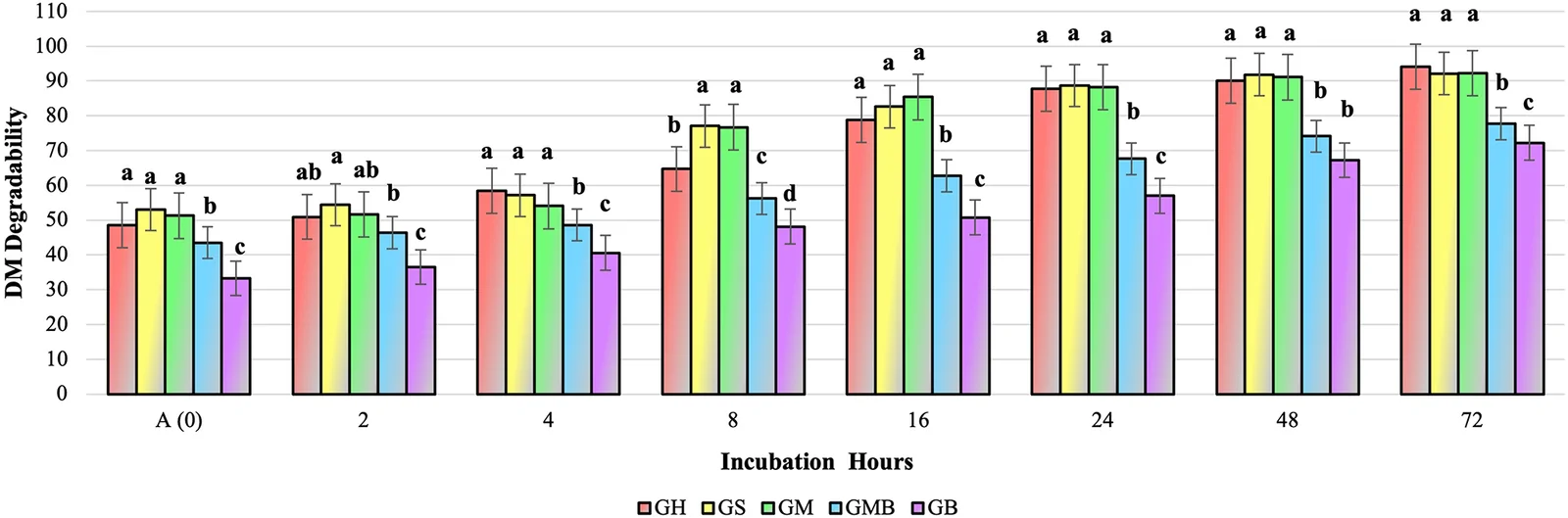
DM degradability of feed samples according to incubation hours (%). ^a, b, c^The difference between values carrying different letters in the same column was found to be statistically significant, (*P* < 0.05), *n* = 2 biological replicates; each value is the mean of three technical replicates per animal, A: Water-soluble fraction of crude protein, washing loss; GH: guar hay; GS: guar silage; GM: guar+ molasses silage; GMB: guar+molasses+straw silage; GB: guar+straw silage

When examining the DM degradability results based on incubation hours presented in Fig. 2, the highest wash loss was observed in the GS group; however, the difference between this group and the GH and GM groups was not statistically significant. The lowest wash loss was identified in the GB group, and this value was significantly lower than that of all other groups. In the early incubation hours (at the 2nd and 4th hours), the highest DM degradability values were typically observed in the GS or GH groups, while the lowest values were consistently recorded in the straw containing GB group, and these differences were found to be statistically significant. At the 8th hour, the highest degradability was again noted in the GS group, and the lowest in the GB group, indicating that the inclusion of straw significantly diminished degradability. During longer incubation periods (at 16, 24, 48, and 72 h), no significant differences emerged among the GH, GS, and GM groups, whereas the straw containing groups (particularly GB) exhibited the lowest values. Overall, it can be concluded that the GS group often demonstrated the highest or high DM degradability values, while the addition of straw led to a significant and statistically meaningful reduction in degradability. Additionally, no notable effect of molasses supplementation on DM degradability was identified.

**Fig. 3:**
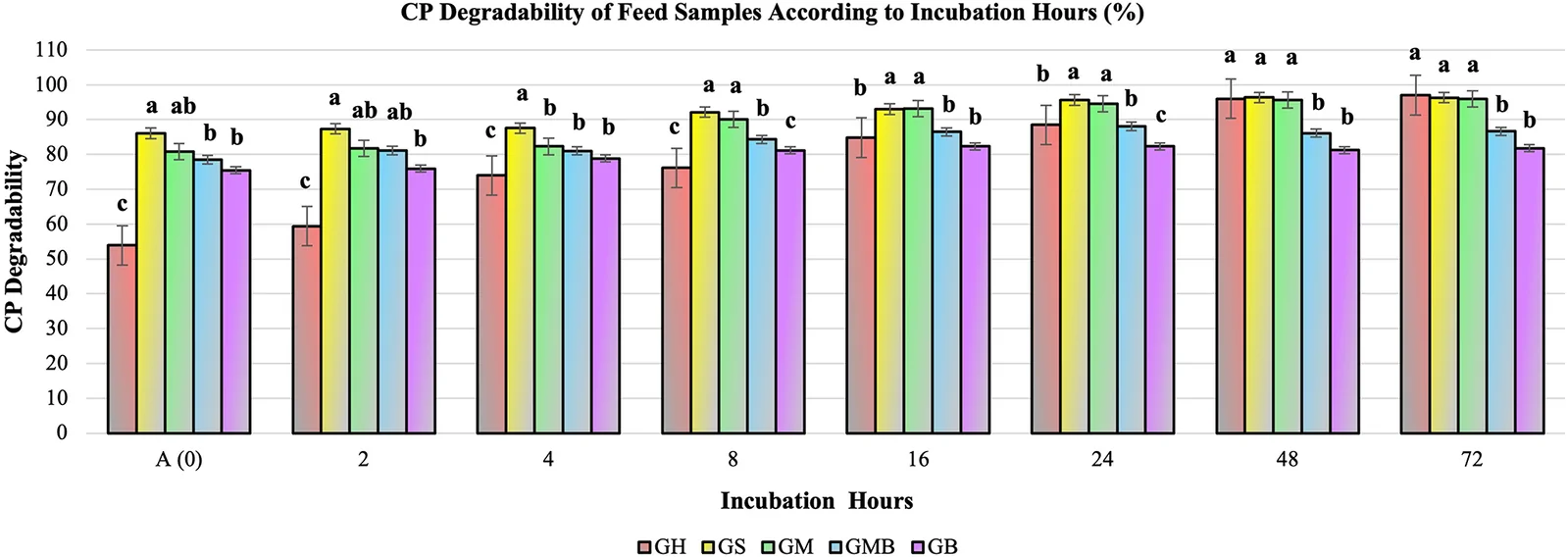
CP degcadability of feed samples according to incubation hours (%). ^a, b, c^The difference between values carrying different letters in the same column was found to be statistically significant, (*P* < 0.05), *n* = 2 biological replicates; each value is the mean of three technical replicates per animal, A: Water-soluble fraction of crude protein, washing loss; GH: guar hay; GS: guar silage; GM: guar+molasses silage; GMB: guar+molasses+straw silage; GB: guar+straw silage

According to the results presented in Fig. 3, the GH group generally exhibited lower CP degradability compared to the silage groups. The additive free guar silage (GS) demonstrated the highest CP degradability values across nearly all incubation hours. The effect of molasses supplementation (GM) on degradability was limited, and no significant difference was observed, particularly between GS and GM. The inclusion of straw decreased protein degradability significantly, both when used alone (GB) and in combination with molasses (GMB). The silage groups without straw (GS and GM) showed higher degradability compared to those containing straw. As the incubation period increased, protein degradability rose across all groups; however, the straw-containing groups continued to exhibit lower values compared to the others. Overall, the highest protein degradability values were recorded in the GS and GM groups, while the lowest were particularly observed in the straw-containing GB group. Consequently, it can be concluded that guar silage demonstrates higher degradability in terms of crude protein compared to dry forage.

### Dry matter, organic matter and crude protein degradability parameters of feeds

**Fig. 4:**
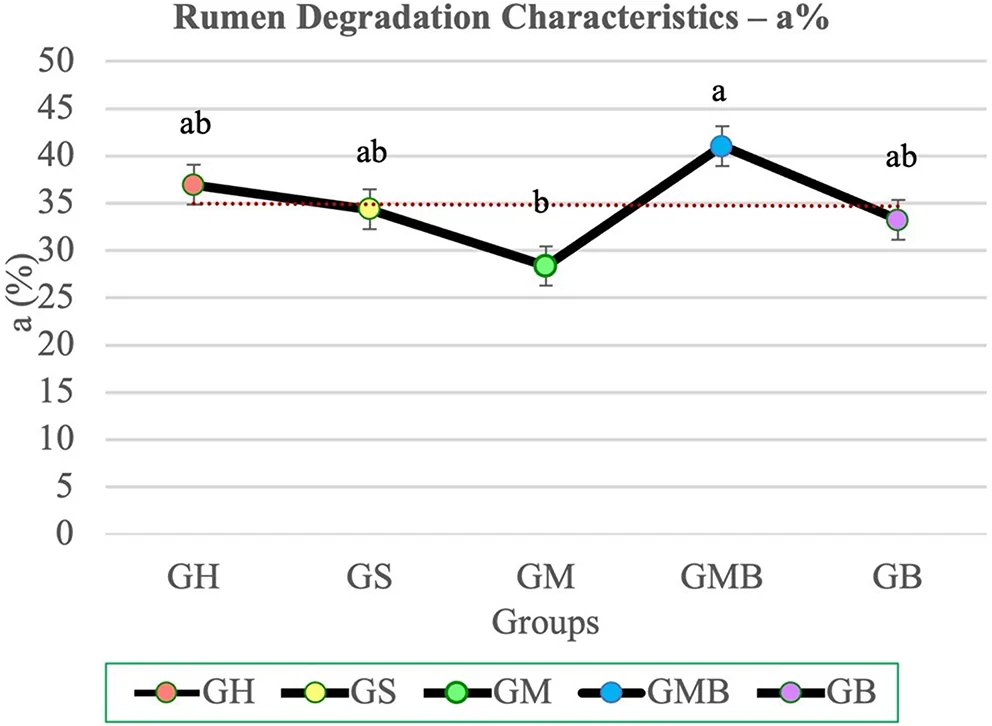
Rumen degradation characteristics – a% for organic matter. a: rumen degradability of rapidly soluble fraction, GH: guar hay; GS: guar silage; GM: guar+ molasses silage; GMB: guar+molasses+straw silage; GB: guar+straw silage; a, b: values with different letters in the same line are statistically significant (*P* < 0.05), *n* = 2 biological replicates; each value is the mean of three technical replicates per animal

**Fig. 5:**
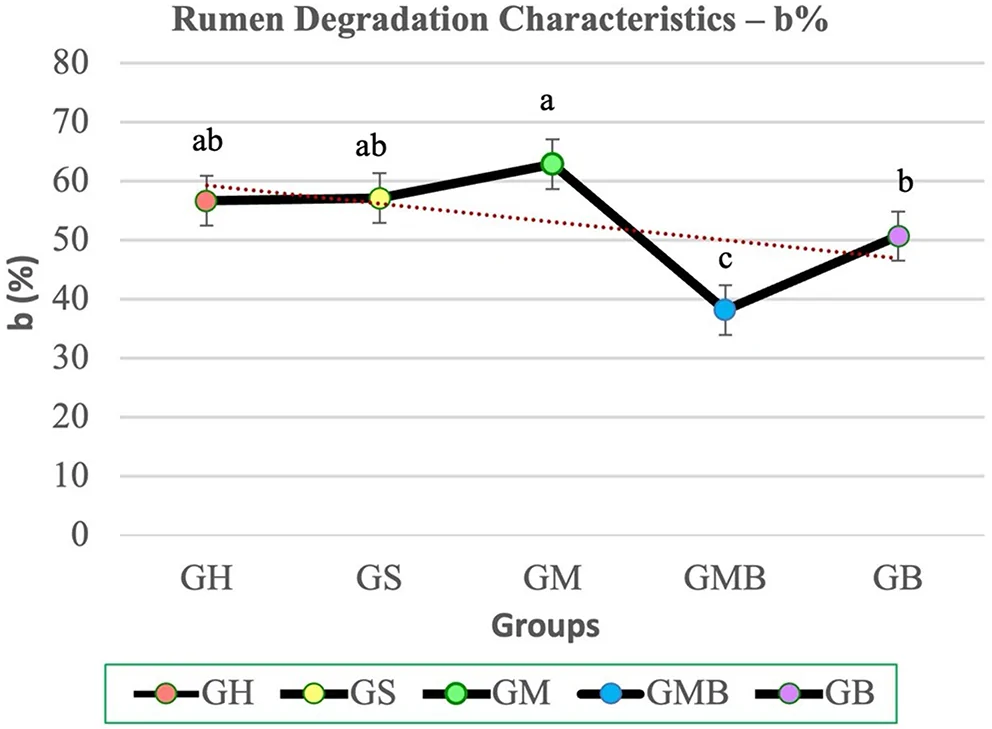
Rumen degradation characteristics – b% for organic matter. b: degradability of the fraction that is non-soluble but degradable over timeGH: guar hay; GS: guar silage; GM: guar+ molasses silage; GMB: guar+molasses+straw silage; GB: guar+straw silage; a, b, c: values with different letters in the same line are statistically significant (*P* < 0.05), *n* = 2 biological replicates; each value is the mean of three technical replicates per animal

**Fig. 6:**
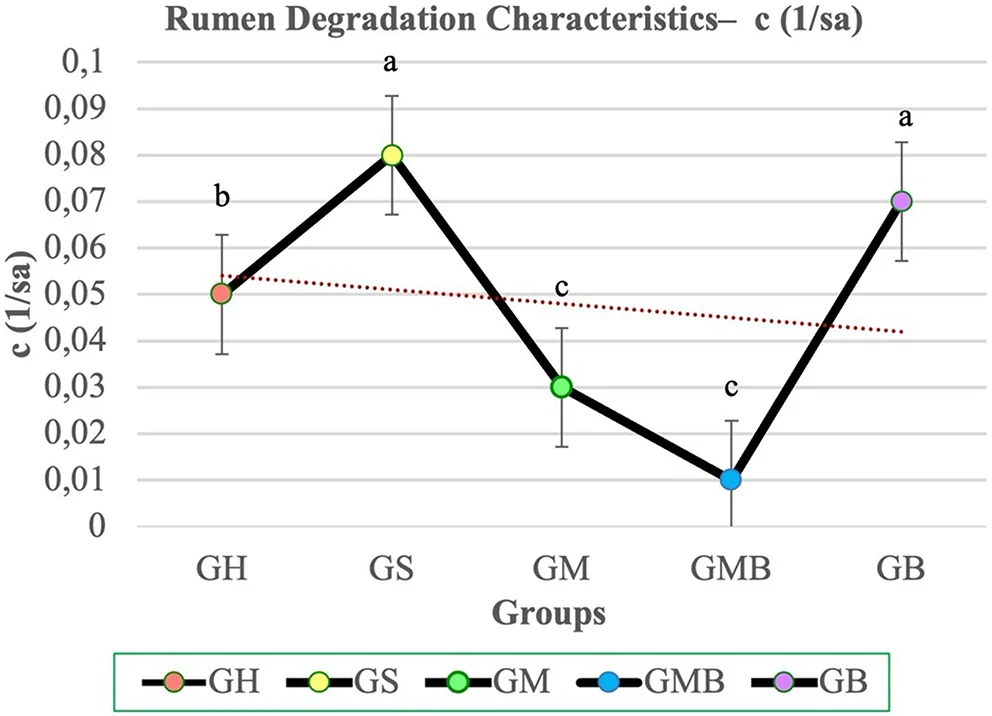
Rumen degradation characteristics– c (1/sa) for organic matter. c: degradation rate constant of b, GH: guar hay; GS: guar silage; GM: guar+ molasses silage; GMB: guar+molasses+straw silage; GB: guar+straw silage; a, b, c: values with different letters in the same line are statistically significant (*P* < 0.05), *n* = 2 biological replicates; each value is the mean of three technical replicates per animal

**Fig. 7:**
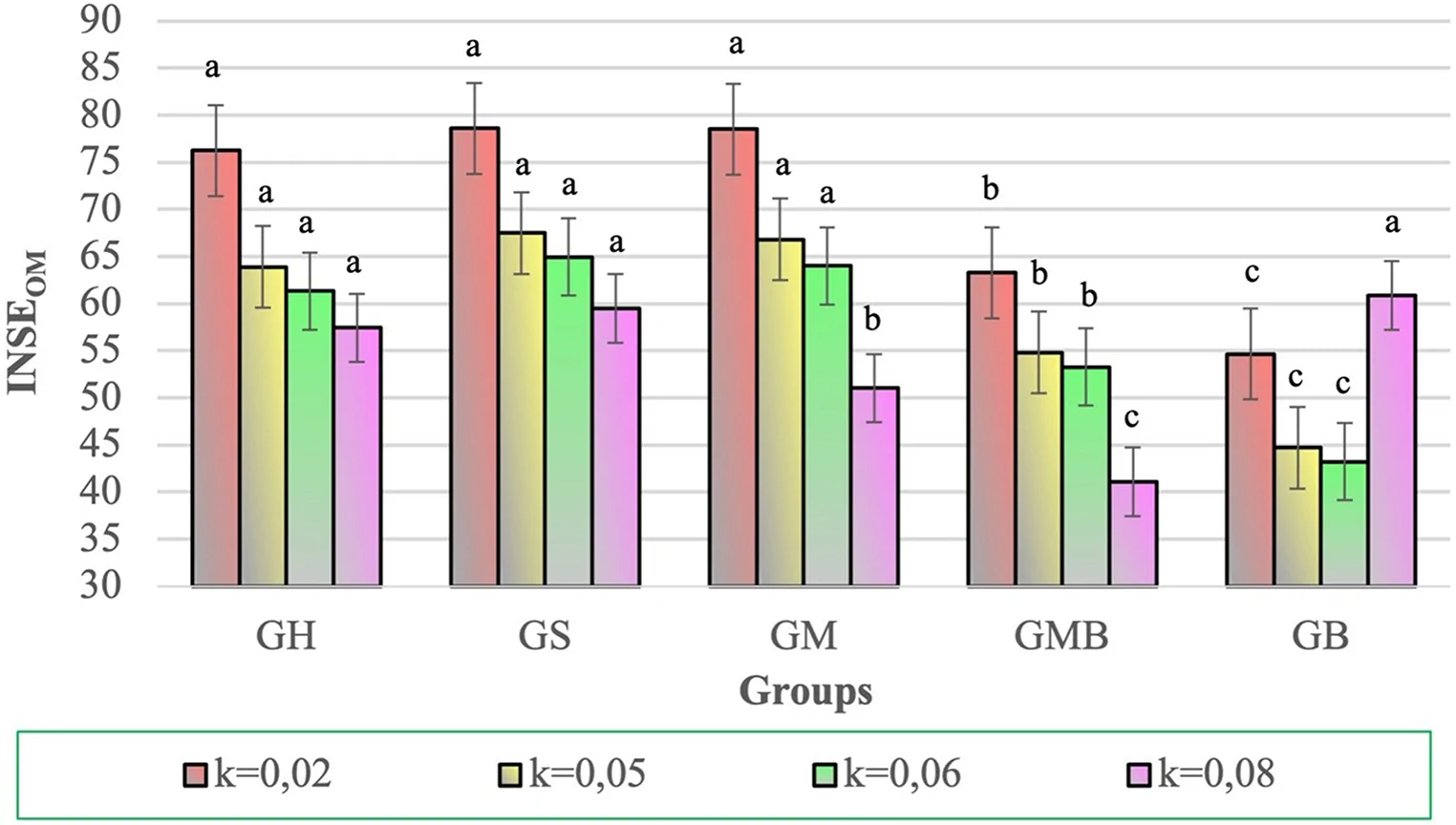
Rumen degradation characteristics–INSE_OM_. INSE_OM_: in situ effective organic matter degradability, k: organic matter passage rate from the rumen, GH: guar hay; GS: guar silage; GM: guar+ molasses silage; GMB: guar+molasses+straw silage; GB: guar+straw silage; a, b, c: values with different letters in the same column are statistically significant (*P* < 0.05), *n* = 2 biological replicates; each value is the mean of three technical replicates per animal

In Situ Rumen Organic Matter Degradability Parameters of Feed Samples, presented in Figs. 4, 5 and 6, and 7, the addition of molasses generally reduced the rate of OM degradation in the rumen. When straw and molasses were used together (GMS group), the soluble fraction and degradation rate values were observed to be at their lowest levels. Although the GM group exhibited the highest time dependent degradation rate, it showed the lowest value in terms of the soluble fraction. The incorporation of straw significantly reduced OM degradability, particularly in the GB group. Overall, the effective degradability of dry matter for the GH, GS, and GM groups was found to be high, while the lowest value was recorded in the straw-containing GH group. Among the molasses containing groups, GMB demonstrated lower degradability compared to GM. Consequently, it can be concluded that the addition of molasses and, in particular, straw negatively impacts OM degradability, with the best results being obtained from groups containing dry forage and additive free silage.

### In situ rumen organic matter degradability parameters of feed samples

### Parameters of in situ rumen dry matter degradability of feed samples

**Fig. 8:**
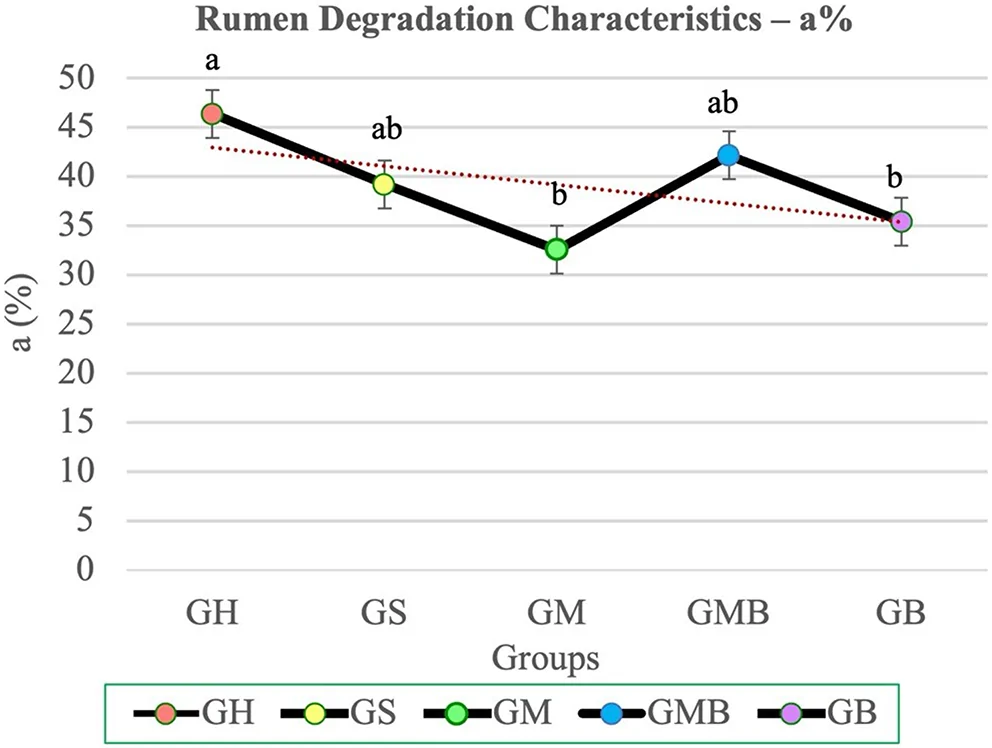
Rumen degradation characteristics – a% for dry matter. a: rumen degradability of rapidly soluble fraction, GH: guar hay; GS: guar silage; GM: guar+melas silage; GMB: guar+molasses+straw silage; GB: guar+straw silage; a, b: values with different letters in the same line are statistically significant (*P* < 0.05), *n* = 2 biological replicates; each value is the mean of three technical replicates per animal

**Fig. 9:**
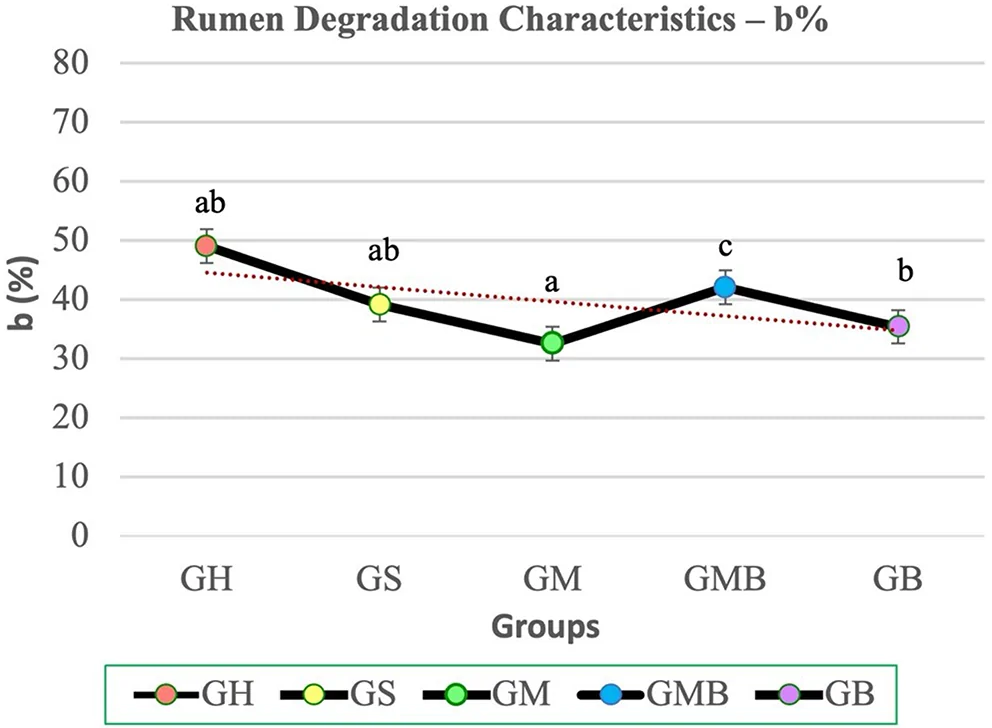
Rumen degradation characteristics – b% for dry matter. b: degradability of the fraction that is non-soluble but degradable over time GH: guar hay; GS: guar silage; GM: guar+ molasses silage; GMB: guar+molasses+straw silage; GB: guar+straw silage; a, b, c: values with different letters in the same line are statistically significant (*P* < 0.05), *n* = 2 biological replicates; each value is the mean of three technical replicates per animal

**Fig. 10:**
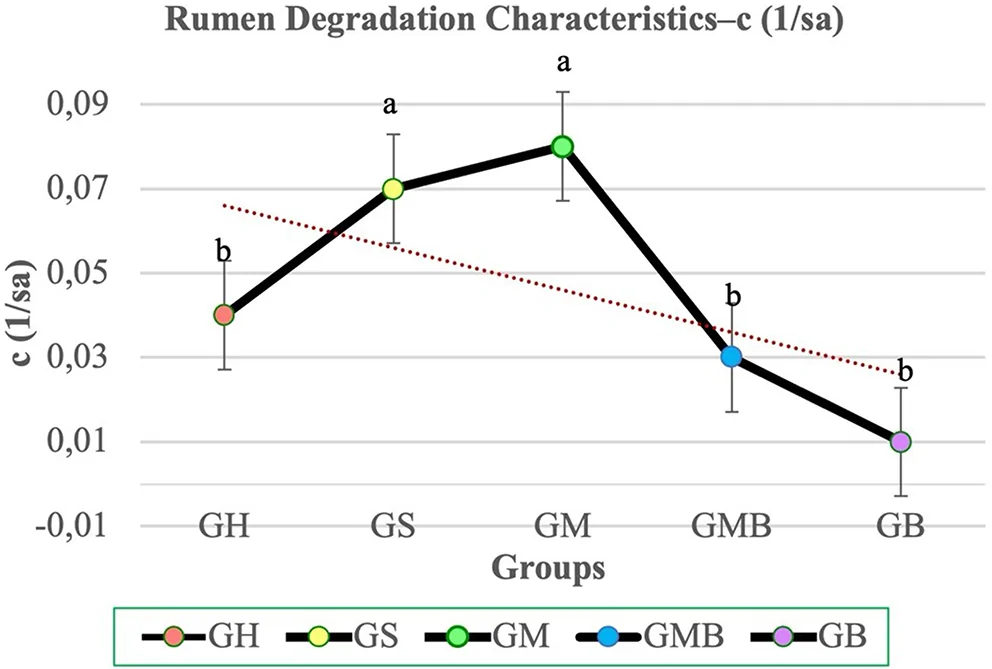
Rumen degradation characteristics– c (1/sa) for dry matter. c: degradation rate constant of b, GH: guar hay; GS: guar silage; GM: guar+melas silage; GMB: guar+molasses+straw silage; GB: guar+straw silage; a, b, c: values with different letters in the same line are statistically significant (*P* < 0.05), *n* = 2 biological replicates; each value is the mean of three technical replicates per animal

**Fig. 11:**
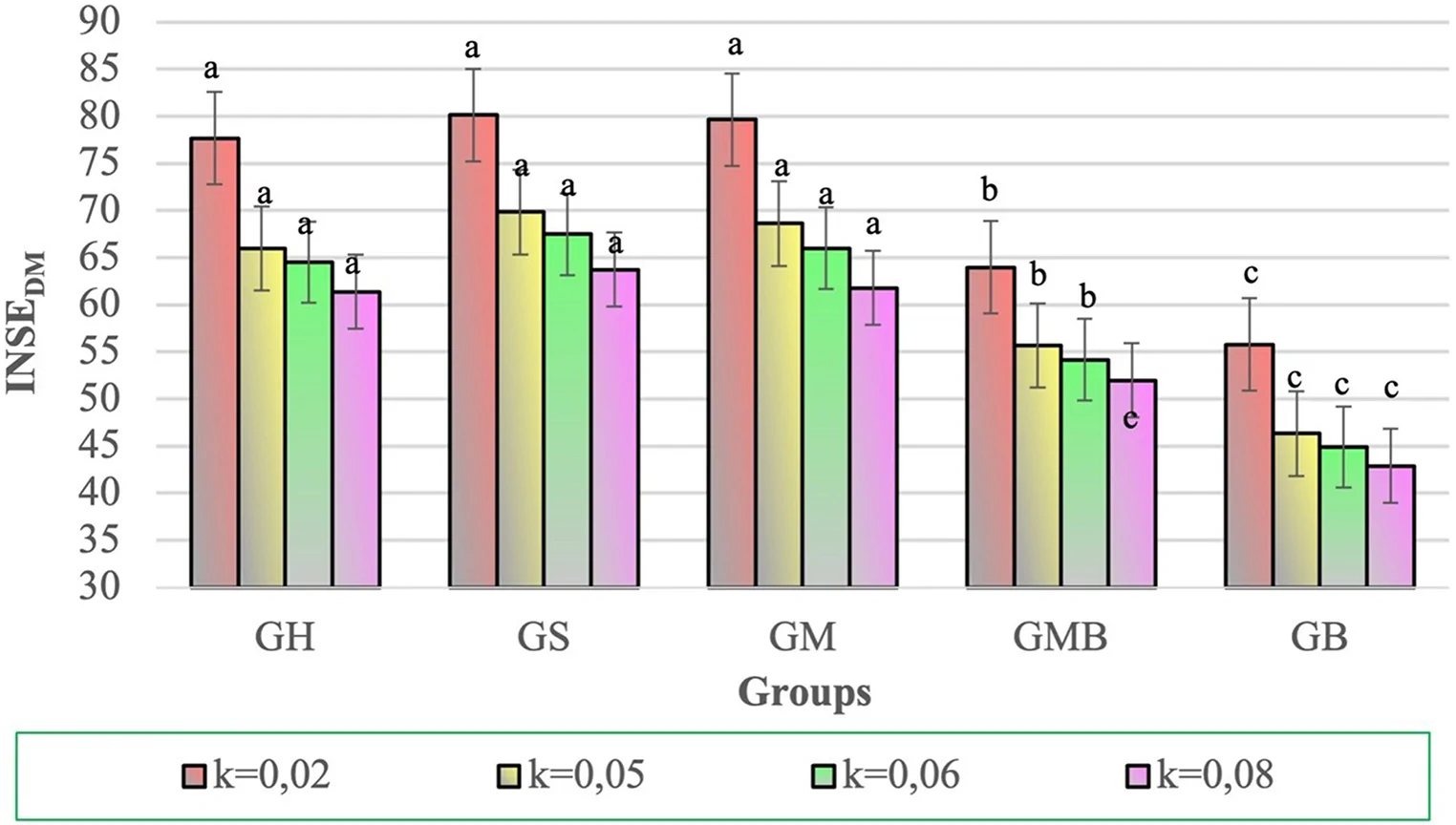
Rumen degradation characteristics–INSE_DM_. INSE_DM_: in situ effective dry matter degradability, k: dry matter passage rate from the rumen, GH: guar hay; GS: guar silage; GM: guar+ molasses silage; GMB: guar+molasses+straw silage; GB: guar+straw silage; a, b, c: values with different letters in the same column are statistically significant (*P* < 0.05), *n* = 2 biological replicates; each value is the mean of three technical replicates per animal

According to the results presented in Figs. 8, 9, 10 and 11, the immediately soluble DM fraction was highest in the GH group and lowest in the GM group. The highest time dependent degradability was observed in the GM group, while the GMB group exhibited the lowest value. In terms of degradation rate, GM showed a prominent performance, whereas GB was identified as the slowest degrading feed. Regarding INSE_DM_ values, the highest value was recorded in the GS group and the lowest in the GB group, with this decline found to be statistically significant (*p* < 0.05). Overall, the addition of molasses appeared to enhance the digestion rate, while the incorporation of straw significantly reduced digestibility.

### Parameters of in situ rumen crude protein degradability of feed samples

**Fig. 12:**
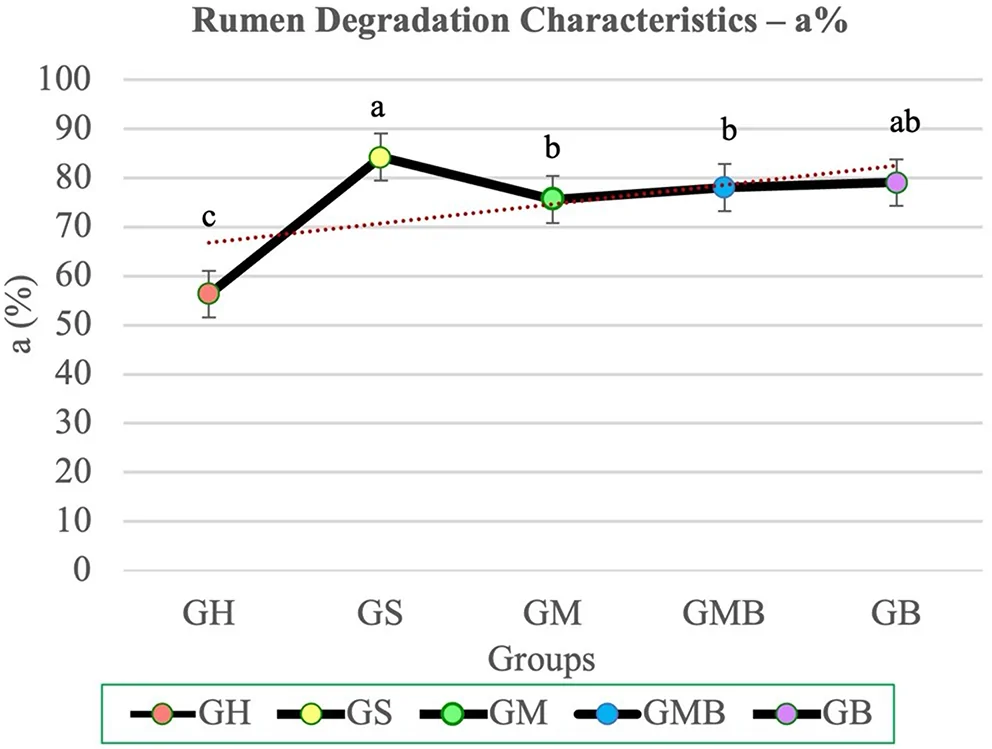
Rumen degradation characteristics – a% for curude protein. a: rumen degradability of rapidly soluble fraction, GH: guar hay; GS: guar silage; GM: guar+melas silage; GMB: guar+molasses+straw silage; GB: guar+straw silage; a, b, c: values with different letters in the same line are statistically significant (*P* < 0.05), *n* = 2 biological replicates; each value is the mean of three technical replicates per animal

**Fig. 13:**
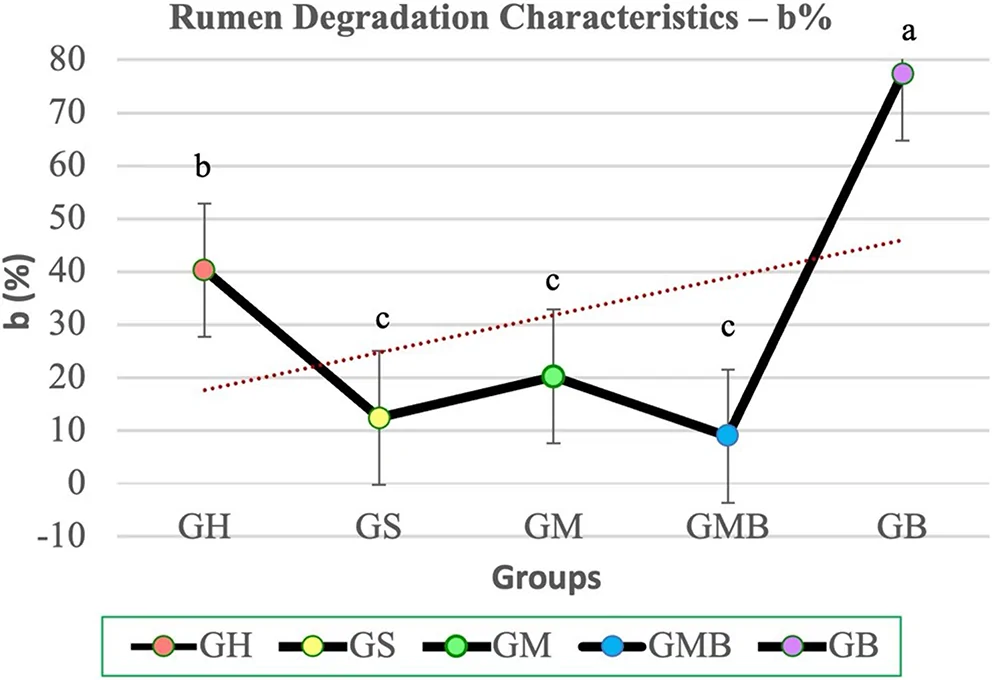
Rumen degradation characteristics – b% for curude protein. b: degradability of the fraction that is non-soluble but degradable over time GH: guar hay; GS: guar silage; GM: guar+ molasses silage; GMB: guar+molasses+straw silage; GB: guar+straw silage; a, b, c: values with different letters in the same line are statistically significant (*P* < 0.05), *n* = 2 biological replicates; each value is the mean of three technical replicates per animal

**Fig. 14:**
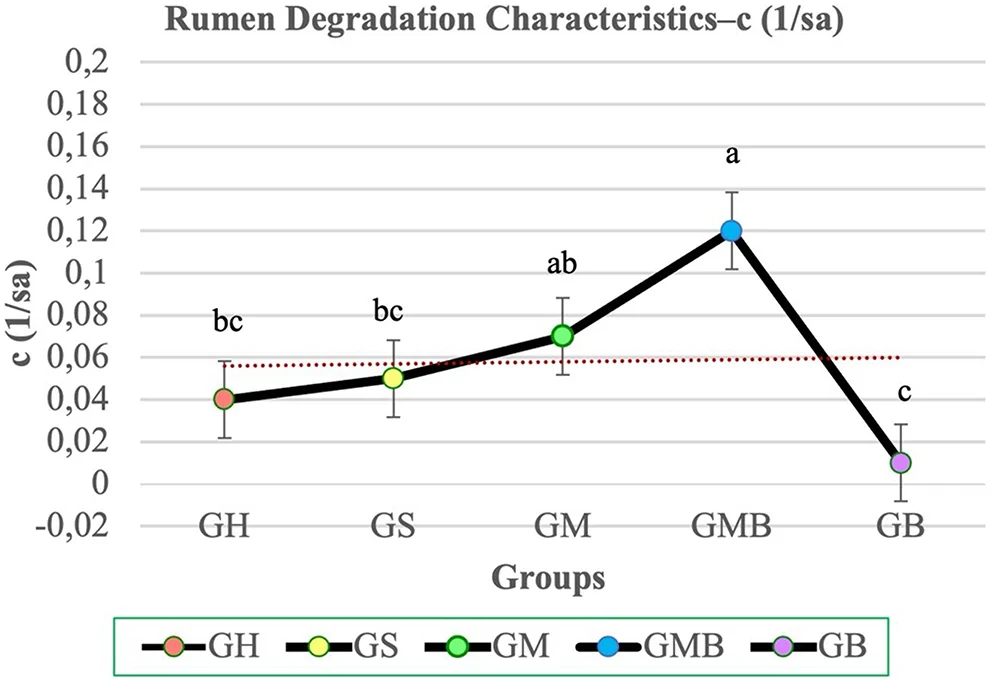
Rumen degradation characteristics– c (1/sa) for curude protein. c: degradation rate constant of b, GH: guar hay; GS: guar silage; GM: guar+melas silage; GMB: guar+molasses+straw silage; GB: guar+straw silage; a, b, c: values with different letters in the same line are statistically significant (*P* < 0.05), *n* = 2 biological replicates; each value is the mean of three technical replicates per animal

**Fig. 15:**
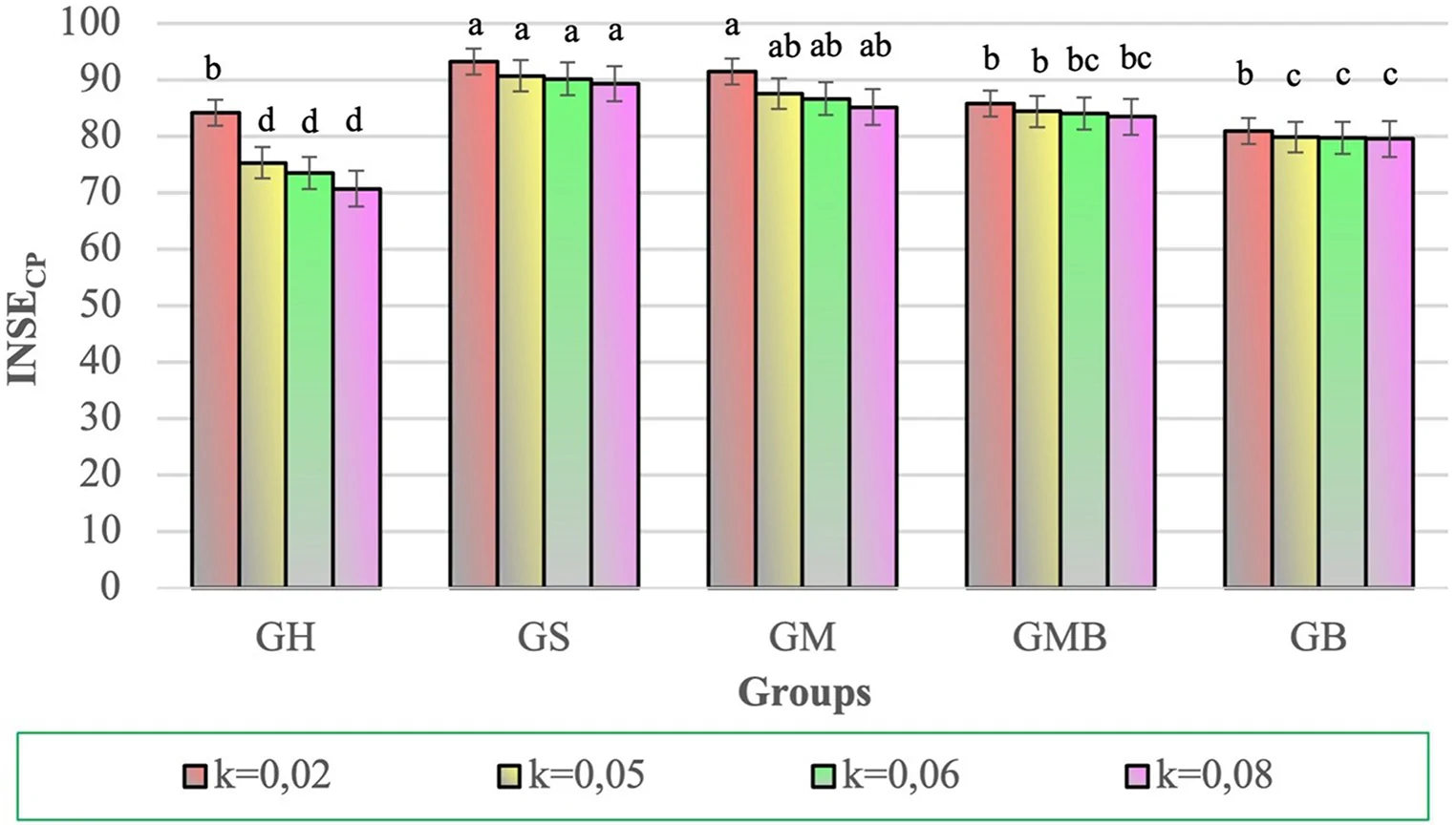
Rumen Degradation Characteristics–INSE_CP_. INSE_CP_: in situ effective crude protein degradability, k: protein passage rate from the rumen, GH: guar hay; GS: guar silage; GM: guar+ molasses silage; GMB: guar+molasses+straw silage; GB: guar+straw silage; a, b, c,d: values with different letters in the same column are statistically significant (*P* < 0.05), *n* = 2 biological replicates; each value is the mean of three technical replicates per animal

According to the results presented in Figs. 12, 13, 14 and 15 the GH group exhibited the lowest value for the immediately soluble CP fraction in the rumen. In contrast, GS group reached the highest values in this regard, although the difference between GS and the GB group was not statistically significant. When evaluating the time dependent degradable fraction (b), the highest ratio was found in the GB group, while the lowest ratio was observed in the GMB group, which included a combination of straw and molasses. Among the molasses containing groups, similar low b values were noted, comparable to those of the additive-free silage. In terms of the degradation rate constant, the fastest CP degradation was observed in the straw containing GB group, while the slowest degradation was again identified in some silage groups. The inclusion of molasses did not result in a significant improvement in degradation rates. The effective CP degradability (INSE_CP_) values calculated based on the outflow rate from the rumen showed the highest result in the GS group and the lowest in the GB group. Generally, the INSE_CP_ values of the silage groups were higher compared to GH, and this difference was statistically significant (*p* < 0.05). In conclusion, the guar silage groups, particularly GS, demonstrated greater ruminal crude protein degradability compared to GH; however, the addition of straw negatively impacted this value. The effect of molasses addition was limited.

## Discussion

### Nutrient composition

In this study, GS and GM silages produced under vacuum-sealed laboratory conditions had relatively high DM and CA but low EE, CF, and fiber fractions (NDF, ADF, ADL) compared with typical forage silages.

Olfaz et al. (2019) reported lower DM (25.50–31.24%) and CA (8.67–9.28%) but higher EE, CF, NDF, ADF, and ADL contents in GS and GM silages than those observed in the present study. Their CP values ranged from 13.88% to 21.64%, with our GS results being slightly higher and GM results slightly lower. These differences are likely attributable to variations in DM content at harvest (27.58% in Olfaz et al. vs. 32.45% in the present study), molasses inclusion rates (10% vs. 5%, respectively), regional climatic conditions, and potential agronomic or genetic differences in plant materials. In particular, the lower molasses rate and relatively later harvest period in the present study may have influenced fermentation dynamics and, consequently, the nutrient composition of the silages.

Amasaib et al. (2016b) evaluated seven guar genotypes for their potential as ruminant feed and reported significant variation in chemical composition (CP, CF, EE, CA, NDF, ADF, and ADL; *P* < 0.01). CP content ranged from 28.74% to 23.82%, while other parameters such as EE, CF, and fiber fractions showed considerable differences among genotypes. Metabolizable energy values varied between 8.51 and 9.35 MJ/kgDM. Compared with their findings, the guar hay (GH) used in the present study exhibited slightly higher CP but lower CA, NDF, ADF, ADL, and ME values, whereas CF and EE contents were comparable. These discrepancies may stem from differences in geographical and climatic conditions, cultivation practices, and harvest time; notably, Amasaib et al. did not provide details on cultivation conditions.

Kuşvuran et al. (2019) highlighted that guar, a leguminous species, is a significant plant with the potential to meet the roughage needs of livestock and holds considerable value for cultivation in Turkey. In their study conducted in Çankırı province, they aimed to determine the effects of different harvesting times (early vegetative stage, beginning of flowering, 50% flowering, full flowering, end of flowering, beginning of pod formation, full pod formation, and the period when seeds begin to form in the pods) on the forage yield and quality of guar. The results indicated that the green forage yield ranged from 838 to 3874 kg da⁻¹, dry forage yield from 252 to 989 kg da⁻¹, CP content from 16.2% to 19.8%, CF content from 48.5% to 55.0%, ADF from 38.7% to 42.9%, and NDF from 43.5% to 49.8%. According to the findings of the study, the most suitable harvesting time in terms of forage yield and quality was concluded to be at the end of flowering or at the beginning of pod formation. In our study, the CP content of the guar harvested at 75 days was detected to be 15.84%, a value close to that of the guar plant group harvested at 50% flowering reported by Kuşvuran et al. (2019). This 75-day period is characterized by ongoing flowering, with the pods not yet having begun to fully mature. The CF, NDF, and ADL values found by Kuşvuran et al. (2019) were observed to be higher than those obtained in our research (19.61%, 19.72%, and 17.64% respectively).

Alaca and Parlak (2017) reported a CP content of 11.93% and CA, NDF, ADF, and ADL values of 9.02%, 43.70%, 21.54%, and 2.26% for guar silage. In the present study, GS exhibited higher CP and CA, lower NDF and ADF, and a comparable ADL content. These differences may be explained by variations in climatic conditions and harvest times between the studies.

Suliman et al. (2017) reported that the CP content of guar silage was 14.60%, while green guar forage contained 15.42% CP. In our study, guar hay showed a similar CP level (15.67%), but had higher OM (88.77% vs. ~85%) and lower CF (17.63% vs. ~24%), EE (2.19% vs. ~2.4%), and CA (11.23% vs. ~15%). Such differences may arise from variations in harvest time, environmental conditions, and preservation methods.

Gökkaya and Orak (2021) conducted a two-year study under arid conditions in Tekirdağ to examine the yield and quality traits of different alfalfa (*Medicago sativa* L.) cultivars harvested at two flowering stages (10% and 50%). Green herbage yield at 50% flowering was approximately 4,350 kg ha⁻¹, which was higher than at 10% flowering (about 3,190 kg ha⁻¹). Crude protein content remained around 21% at both stages. ADF values were recorded in the range of 40.15–42.47% in the first year and 39.60–43.25% in the second year, indicating a decline in digestibility with advancing maturity. NDF values ranged from 40.96 to 43.00% in the first year to 39.95–47.22% in the second year, reflecting variations in potential feed intake. In comparison, Kuşvuran et al. (2019) reported green herbage yields for guar (*Cyamopsis tetragonoloba* L.) between 838 and 3,874 kg ha⁻¹, which are lower than those for alfalfa; however, given the limited options for summer forage legumes in Turkey, guar represents a significant alternative. Incorporating species adapted to local conditions into forage production systems, as emphasized by Abbasi (2025), is of strategic importance for enhancing the resilience of livestock enterprises in regions where climatic variability threatens forage resource sustainability.

To facilitate comparisons with guar bean, a review of the literature was conducted regarding soybean (*Glycine max L.*), an annual legume that can be cultivated as either a cool or warm season crop. Soybean is not only important as a raw material for grain or meal production but also serves as a significant source of forage for ruminants. In their study, Ergin and Aydemir (2018) concluded that when evaluating soybeans for silage, it is more effective to grow them in mixtures with certain warm-climate cereals, such as corn, sorghum, or Sudan grass, rather than using them in pure stands. Furthermore, Özel and Acar (2020) reported that the green forage yield of the soybean varieties used in their research ranged from 2063.00 to 2837.67 kg/da, while the dry forage yield varied between 904.03 and 1128.27 kg/da. These values fall within the range of green forage yields reported for guar bean (838–3874 kg/da) by Kuşvuran et al. (2019). To enable a more accurate comparison of yield parameters for these two plants (soybean and guar) in ruminant nutrition, further studies are required.

### Saponin contents of feed samples

Seven guar genotypes cultivated for forage production were used in the study conducted by Amasaib et al. (2016a), in which the genotype was reported to have a highly significant effect on saponin content (*P* < 0.01). In those guar hay samples, saponin concentrations ranged from 0.1147% to a maximum of 0.7008%. In our study, the values determined were 3.82% GAE for GH and 3.96% GAE for GS. The relatively low results obtained by Amasaib et al. (2016a) from guar hay samples, compared with the higher GAE percentages in our study, may primarily be attributed to the analytical enrichment (concentration) steps applied.This likely reflects differences arising both from the semi‑quantitative nature of our methodology and from the intrinsic potential of the guar material analyzed.

Although the extraction and re‑dissolution steps we employed appeared to successfully reveal the saponin potential in guar hay, our results are classified as semi‑quantitative rather than absolute quantitative values. This methodological limitation can be explained under two main points. First, guar bean saponins belong to the triterpenoid saponin class, which also predominates in soybean and alfalfa. Glycyrrhizic acid, derived from licorice root and structurally a triterpene glycoside, was therefore selected as an external reference standard due to its belonging to the same general chemical class (triterpenoid) as guar saponins (Lásztity et al. 1998; Sezgin and Artik 2010). Second, since guar saponins possess a bidesmosidic structure (with two sugar chains) and glycyrrhizic acid is not structurally identical, and it is therefore not expected to elicit the same response in HPLC analysis (Curl et al. 1986; Hassan 2008; Hassan et al. 2010). Triterpene bidesmosides are known to have lower hemolytic activity compared with monodesmosides (Sezgin and Artik 2010; Hassan et al. 2010). In this context, considering the natural diversity of saponin profiles among different guar genotypes and the characteristics of the analytical methods used, deviations of our GAE results from the ranges reported by Amasaib et al. (2016a) are to be expected.

Nevertheless, the literature clearly indicates that the saponin content of guar (*Cyamopsis tetragonoloba*) can be highly variable (Hassan 2008; Hassan et al. 2013). This variability arises from genetic factors, environmental conditions, and post harvest processing. Several researchers have reported that, in addition to the analytical methods employed, other factors such as year of cultivation, production site, and season can also influence saponin levels (Oleszek, 1999; Önning et al. 1993; Hassan 2008). It has been reported that guar meal, used in human and animal nutrition, contains crude saponins at levels ranging from 5% to 13% of its dry weight (Hassan 2008). Although the materials we analyzed (hay and silage) do not correspond to this by‑product, such data support the view that the guar species inherently possesses a high saponin potential. Although the high saponin GAE values obtained in this study (3.82–3.96%) reflect guar’s inherently elevated saponin content, the known antinutritional effects of saponins including reduced feed palatability and adverse impacts on animal metabolism and digestive efficiency (Mahala et al. 2012; Salama and Nawar 2016) efficiency suggest that saponin content may constitute a limiting factor for the practical use of guar as a roughage.

### Dry matter digestibility, dry matter consumption and relative feed values

The Relative Feed Value is a composite index designed to estimate forage quality by integrating predicted DMI and dry matter digestibility (DMD), both derived from fiber fractions. The RFV scale is standardised against alfalfa hay at full bloom (41% ADF, 53% NDF, RFV = 100) and is frequently used by researchers and the forage industry to classify feed into quality categories (Moore and Undersander 2002; Tremblay 1998).

In our study, non-straw groups exhibited RFV values between 321.81 and 354.51 (GH, GS, GM), substantially above the highest quality class threshold (> 150). Groups containing straw showed markedly lower RFV values: GMB = 139.12 (1st quality) and GB = 109.21 (2nd quality). However, this classification is based on an index calculated from fiber fractions and is not supported by animal performance data.

Similar trends have been reported in the literature, but generally within lower numerical ranges. For example, Oflaz et al. (2019) found GS and GM to have RFV values of 128.98 (2nd quality) and 174.14 (1st quality), respectively; Amasaib et al. (2016a) reported GH genotypes ranging between 128.13 and 159.06; and Kuşvuran et al. (2019) recorded maximum RFV values of 120.8 during flowering. The comparatively high RFV values in the present study can be directly attributed to notably low NDF and ADF contents observed in certain treatments.

Low fiber fractions are known to elevate RFV mathematically and physiologically. Reduced cell wall content enhances potential intake and digestibility. This is often achieved in early harvest stages or with leguminous species of high nutritive value (Buxton and Redfearn 1997; Jeranyama and Garcia 2004). In our case, the non-straw groups likely contained plant material with less developed cell walls and lower lignification, resulting in lower NDF and ADF values and consequently elevated RFV.

These findings reinforce the relationship between fiber composition and RFV and illustrate how cutting stage, plant morphology, and inclusion of fibrous additives (e.g., straw) markedly affect this metric. This study adds to existing knowledge by demonstrating that guar, under certain harvest conditions and without coarse fiber additives, can achieve substantially elevated RFV values.

### Organic acid levels

Upon reviewing the relevant literature, notable differences were identified between the findings of the present study and those reported by Oflaz et al. (2019). In their study, the control guar silage group without additives exhibited LA, AA, PA, and BA levels of 3.45%, 2.23%, 2.67%, and 5.50%, respectively, whereas the GM contained 11.3% LA, 3.24% AA, 0.96% PA, and 0.02% BA. In our study, the GS group showed a higher LA content (5.58%), a similar AA level (2.42%), and lower BA (0.54%) and PA (0.00%) concentrations compared to the GS reported by Oflaz et al. (2019). Interestingly, Oflaz et al. observed LA levels in GS to be lower than BA levels, while our data revealed the opposite pattern, suggesting that the GS silage in the present investigation was of comparatively higher fermentation quality in terms of organic acid composition. Furthermore, when comparing guar silages with molasses addition, Oflaz et al. (2019) reported 11.30% LA, 1.92% AA, 0.07% PA and 1.00% BA on a dry matter basis; these values are markedly higher than the LA (1.30%) and other acid levels observed in our GM group. Several factors may account for these discrepancies, including differences in plant harvest stage, nutritional composition, and the quantity of molasses added; notably, Oflaz et al. (2019) utilized a 10% molasses inclusion rate, whereas our study employed 5%. The latter was selected as an optimal balance to enhance fermentation quality, limit proteolysis, and reduce effluent losses, while maintaining economic feasibility (Mordenti et al. 2021). Nevertheless, altering molasses levels can markedly influence microbial dynamics during fermentation, resulting in distinct organic acid profiles. Therefore, molasses inclusion rates should be determined with careful consideration of fermentation performance, economic sustainability, and the intended end-use of the silage. There is a significant relationship between the proportions of LA%, AA%, and BA% and the quality of silage. A quality silage typically has a lactic acid level exceeding 2%, whereas the ideal range for AA is between 0.3% and 0.7%. In high-quality silage, the presence of BA is generally undesirable and averages between 0.1% and 0.6% (Kılıç 1986). In our research, the lactic and BA contents of the GMB and GS groups were within acceptable levels for quality silage; however, the AA content was found to be above the recommended threshold.

The fermentation of feed plants is a complex process wherein various types of microorganisms can lead to the production of different end products. During the initial phase of silage fermentation, different microbial groups capable of anaerobic growth (including lactic acid bacteria, enterobacteria, clostridia, and yeasts) compete for the nutrients present in the silage. In well-preserved silages, lactic acid bacteria rapidly become dominant, leading to a decrease in pH and a reduced formation of acetic acid due to the accumulation of lactic acid (Driehuis and Wikselaar 2000). A rapid reduction in pH is favorable as it diminishes the prevalence of enterobacteria, which could otherwise lead to excessive protein degradation and reduce the risk of undesirable fermentation processes associated with clostridia.

### Silage fermentation characteristics

To achieve high quality silage, it is essential to maintain an acidic environment within the silo, characterized by a low pH and a high production of LA. This can be facilitated by the presence of readily soluble carbohydrate sources, alongside ensuring an adequate protein content and that the feed contains at least 30–35% DM (Demirel and Yıldız 2001). According to Başkavak (2008), the fermentation processes occurring during the silage making period significantly influence various properties of the silage, including DM, pH, organic acid composition, and NH_3−_N levels. These factors have been shown to have substantial effects on dry matter intake and the nutritional value of the silage (Kılıç 1986; McDonald et al. 1988; Yurtman et al. 1997).

### pH levels

A review of the literature reveals that in a study conducted by Oflaz et al. (2019), the pH level of the control group represented by GS, which contained no additives, was found to be 5.90. In contrast, the silage group with molasses addition had a pH 4.62. However, in our research, the pH values of the silage groups were determined to be within the range of 8.00 to 5.74, which are significantly higher than the pH levels reported by Oflaz et al. For high quality silage, an ideal pH range is generally between 3.5 and 4.0. However, pH values of 4.0 and above are frequently observed in legume silages (Filya 2001).

An increase in pH during silage fermentation is observed when lactic acid production is insufficient and proteolytic fermentation becomes dominant (Kung and Shaver 2001). In the GM group, the lack of fermentation proceeding towards lactic acid production may have resulted in the conversion of amino acids into ammonia and amines. The resulting ammonia exerts a buffering effect, causing the pH of the environment to rise above the expected range.

### Aerobic stability

Aerobic stability is a period used by nutritionists to define the length of time that silage remains unspoiled and does not heat up after exposure to air (Kung 2010). Technically, this period is expressed as the time elapsed until the silage temperature rises 2 °C above ambient temperature (Wilkinson and Davies 2013). During the fermentation phase, oxygen ingress is relatively limited; however, substantial oxygen entry occurs particularly during the opening and unloading of the silo. This oxygen influx initially activates yeasts that degrade lactic acid, followed by molds and other aerobic microorganisms, thereby initiating a “domino effect” leading to silage spoilage (Kung 2010). As a result of this biochemical process, an increase in temperature, a rise in pH, dry matter losses, and the production of CO₂ occur (Wilkinson and Davies 2013; Çayıroğlu et al. 2016).

In our study, the low O₂ production of 0.36 g/kg DM observed in the GM and GS groups indicates that these groups possess high aerobic stability. In the literature, CO₂ production is recognized as a direct indicator of aerobic deterioration (Filya 2004; Wilkinson and Davies 2013). For example, some researchers have defined silages producing less than 10 g/kg DM of CO₂ over a 5 day period as being aerobically stable (Wilkinson and Davies 2013). Similarly, studies conducted on maize silages have reported CO₂ production values ranging between 4.1 and 38.8 g/kg DM depending on the stage of maturity (Filya 2004). The values obtained in our study are considerably below these threshold levels reported in the literature, demonstrating that the applied treatments were effective in preserving the hygienic quality of the silage and minimizing nutrient losses.

### NH_3−_N levels

The ammonia level in silage is an expression of the water soluble CP content present in silages. A review of the literature reveals that in a study conducted by Oflaz et al. (2019), the NH_3−_N level in GS, which served as the control group and contained no additives, was found to be 3.47% of total nitrogen (%TN). Conversely, the silage group with molasses addition exhibited an NH_3−_N level of 6.69% TN.

When comparing our research findings to those reported by Oflaz et al. (2019), it is observed that the NH_3−_N levels in the GS group (0.14% TN) and the GM group (0.25% TN) in our study were lower. The methodology employed in determining NH3-N levels may have contributed to variability in the results. Differences in analytical techniques or sampling procedures could have led to inconsistencies in the reported ammonia levels. According to Başkavak (2008), it is noted that NH_3−_N levels in high-quality silage should be below 80 g/kg of total nitrogen (Petterson 1988). Furthermore, Carpintero et al. (1979) indicated that an ammonia value below 11% of total nitrogen is characteristic of silages classified as high quality. The results of our study fall within the ranges reported by these researchers.

The elevated pH values observed in the GM group, in conjunction with the increase in NH₃_−_N levels identified within the same group, suggest that the fermentation process is predominantly directed towards proteolytic fermentation, rather than facilitating lactic acid production. In silage environments, proteolytic microorganisms and clostridial species break down raw material proteins and amino acids, leading to the production of NH₃ and amines (Kung and Shaver 2001). As ammonia is a basic compound, it raises the pH by reducing the acidity of the fermentation fluid. Consequently, in this GM group, where lactic acid production is low, it is posited that the formation of basic ammonia may have contributed to the pH values reaching levels as high as 8.0. The elevated NH₃_−_N concentration suggests that proteolytic fermentation is dominant and that the necessary lactic acid bacteria have not proliferated sufficiently. This phenomenon is often observed in materials with high protein content or high buffering capacity, particularly in cases where anaerobic conditions are not established rapidly (Muck 2010). It is suggested that the soluble carbon sources provided by molasses in the GM group did not favor the desired selection for lactic acid bacteria within the microbial population; instead, they may have supported the proliferation of proteolytic microorganisms. Thus, it is proposed that the high pH and elevated NH₃_−_N values observed in our GM group may stem from the same biochemical processes.

### Water soluble carbohydrates

Water soluble carbohydrates serve as substrates for lactic acid bacteria during the ensiling process, enabling the production of organic acids. The primary WSCs present in plants include fructose, glucose, and sucrose (Amer 2010). A limited WSC content can restrict fermentation, while a high WSC content may be utilized by undesirable microorganisms during silage storage (Cherney and Cherney 2003). It has been established that the minimum WSC concentration necessary for successful silage production should range from 20 to 30 g/kg (Wilkinson et al. 1982; Haigh and Parker 1985). If the initial WSC content exceeds 7.0%, it has been noted that this is sufficient for the reduction and stabilization of pH at the end of fermentation (Yang et al. 2006).

In addition, water soluble carbohydrate content is of paramount importance in silage preparation. Its most significant effect is the rapid decrease in pH during the ensiling process (McDonald et al., 1991). According to Amer (2010), the WSC content of silage feed samples is a crucial factor in achieving a decrease in pH and maintaining silage quality. The rapid decline in pH is attributed to the rapid conversion of readily fermentable carbohydrates into lactic acid by lactic acid bacteria (McDonald et al., 1991).

Studies have shown that silages made from plants with high WSC content are of superior quality compared to those from plants with lower WSC content. Specifically, silages derived from plants with high WSC content exhibited lower pH and reduced NH₃-N. Additionally, they had higher lactic acid content compared to those from plants with low WSC levels (Davies et al. 1998; Hassanat et al. 2006; Merry et al. 2006; Downing et al. 2008). However, when the initial WSC content is above 7.0%, further increases in silage quality may be negligible. Consequently, other measures, such as the application of inoculants, may be warranted to improve silage quality when the WSC content of wheat straw exceeds 7.0% (Yang et al. 2006).

In the present study, the WSC content of the GH was measured at 235.62 g/kg DM, a notably high value attributed to the presence of guar gum within the seeds of the guar plant. This high WSC content in guar is mainly due to guar gum predominantly consists of high molecular weight polysaccharides with molecular weights ranging 50,000–8,000,000Da (Roberts 2011). Based on recommendations from other researchers, it is anticipated that the addition of inoculants to the silage groups may positively influence fermentation kinetics and improve silage quality.

### Organic matter degradability and related feed characteristics at different incubation times

To date, no studies have been published in the literature specifically addressing the OM degradability of the guar plant. However, research conducted by Karayilanli and Ayhan (2017) evaluated the OM degradability of alfalfa (*Medicago sativa L.*) across various growth stages. These stages included budding, early flowering, full flowering, and seed setting. Significant differences (*P* < 0.05) in OM degradability were observed across all incubation periods (0–72 h (0, 2, 4, 8, 16, 24, 48, 72 h) and harvest stages. The average degradability values for lucerne across these stages were determined to be 18.64% and 69.27% at 0 and 72 h, respectively; 18.88% and 65.42% at early flowering; 16.53% and 63.17% at full flowering; and 16.21% and 62.18% at the seed setting stage.

When comparing the rumen OM degradability values of alfalfa with those of GH utilized in our study, it was evident that lucerne exhibited lower OM degradability. Additionally, it was observed that the washing loss for GH and other silages derived from guar (GS and GM) was considerably high, with values of 45.47%, 49.46%, and 48.14%, respectively. By the fourth incubation hour, over half of the GH, GS, and GM had degraded in the rumen, with degradation rates of 54.93%, 53.75%, and 51.38%, respectively.

Norton and Poppi (1995) noted that tropical legumes possess low cell wall content and a high proportion of easily digestible, non-lignified mesophilic tissue, which enhances their degradability. According to Amasaib et al. (2016a), the non soluble, time dependent degradable fraction (b) indicates the prolonged presence and slow degradation of particular feeds in the rumen. For instance, feeds characterized by high protein and fiber content are often challenging to digest. It can be expected that feeds with a substantial proportion of slowly degradable fraction will contain the highest fiber content.

This assumption was corroborated in the current study, as silages containing straw (for GMB and GB, the values were 38.21% and 50.72%, respectively) had lower proportions compared to those without straw (for GH, GS, and GM, the values were 56.69%, 57.16%, and 62.93%, respectively). When evaluating the INSE_OM_ values calculated based on the exit rate constants from the rumen for the guar plant and the silage groups created from it, it was observed that, as shown in Fig. 7, the highest values were recorded in the GH, GS, and GM groups. Furthermore, consistent with literature reports, the presence of high cell wall content straw in the study groups resulted in the expected lower INSE_OM_ degradability values for the GMB and GB groups.

### Dry matter degradability and related feed characteristics at different incubation times

In their study, Amasaib et al. (2016b) investigated the DM degradability values of seven different guar genotypes, revealing significant differences across all parameters examined (*P* < 0.01). They reported that substantial differences in rumen degradation among varieties may be attributed to genetic characteristics reflected in significant variability in morphological and chemical compositions (Agbagla-Dohnani et al. 2001). In a 6-hour incubation period, the lowest DM degradability was found for genotype GM1 at 49.8%, while the highest was observed for GM7 at 57.7%. The dry matter degradability for all feed genotypes varied between 65.8% and 71.6% for a 24-hour incubation. The researchers noted that a considerable amount of DM is degraded in the rumen, thereby providing a source of nitrogen that can be utilized for microbial synthesis occurring in the rumen.

When comparing our results with those of Amasaib et al. (2016b), we found higher ruminal DM degradability at all incubation times. This difference likely reflects a greater supply of DM to support microbial synthesis in the rumen. The rapidly degradable fraction (a) for the seven guar feeds ranged from 16% to 34.6%, with genotype GM7 exhibiting the highest value for the rapidly degradable fraction (a). The researchers indicated that this outcome could be attributed to the highest and lowest NDF values recorded for this genotype. In our study, rumen soluble fraction contents of 46.40% in GH and 39.23% in GS were recorded, which are higher than the values reported by Amasaib et al. (2016b). This increase is likely a result of the low NDF contents of the guar samples used in our study, as suggested by the researchers themselves. Similarly, the lower value obtained for the rapidly degradable fraction (a) in the molasses-containing GM group (32.57%) may also be due to the higher NDF content observed in this group.

The time dependent degradable fraction (b) was found by Amasaib et al. (2016b) to range from 31.90% to 53.20% for the seven different genotypes. The values for the GH, GS, and GM groups in our study were measured at 49.09%, 52.85%, and 59.11%, respectively, which are comparable to those reported by Amasaib et al. (2016b). In their study, the rate constant (c) for ruminal degradation varied between 0.01075 and 0.2020, indicating significant differences among the genotypes (*P* < 0.01). In our research, the ruminal dry matter degradation rate constants ranged from 0.01 to 0.08, showing similarities with values identified by other researchers. Our study noted that increased NDF content was associated with a rise in the c values.

The INSE_DM_ value, based on a departure rate constant of 0.08, was found to range between 42.89% and 63.72%, with no statistically significant differences observed among the GH, GS, and GM groups, whereas lower INSE_DM_ rates were noted in the GMB and GB groups containing straw. It is also suggested that the increasing NDF content may have influenced the INSE_DM_ degradability. Amasaib et al. (2016b) reported effective dry matter degradability values for seven different guar genotypes ranging 49.80%–57.80% at a ruminal passage rate of 0.08. In our study, the INSE_DM_ contents in groups without straw addition (GH, GS, and GM) were found to be between 61.37% and 63.32% (at c = 0.08), which are higher than the values reported by Amasaib et al. (2016b), likely indicating the low NDF contents of the feed samples used in our research.

The high degradability in the rumen could be attributed to the combined effects of both proteolytic and cellulolytic enzymes present in the rumen (Alcaide et al. 1996). In a study evaluating silage from soya bean (*Glycine max (L.) Merr. cv. Ryuho*) harvested at two growth stages, Kawamoto et al. (2013) determined that the time dependent degradable DM fraction (b) for whole green soybeans (R6) was 36.01%, while for soybeans with 50% yellowing pods and leaves (R7), the value was 43.3%. They identified the easily fermentable fractions (a) as 40.0% and 33.2%, respectively, with corresponding degradation rate constants (c) of 0.069–0.075. Calculating the effective DM degradability based on a ruminal passage rate of 0.05 yielded values of 60.6% and 59.2%, respectively, for the specified soy samples.

In terms of degradability characteristics in our study for GS, the value for a was found to be 39.23%, which is lower than those determined by Kawamoto et al. (2013) for different soybean maturation stages, while the time dependent degradability value b at 52.85% was higher than that of soybean. When comparing effective ruminal DM degradability values of soybean silages and GS, our measured value of 69.86% (k = 0.05) exceeds those identified by Kawamoto et al. (2013) for various maturation periods of soybean silages. Furthermore, the in vitro DM degradability of thirteen different genotypes of guar evaluated by Das et al. (1975) ranged 62.8%–71.2%. Although the methodology utilized by Das et al. (1975) differed, it can be stated that the findings of our study are comparable to their results.

### Crude protein and associated feed degradability characteristics at different incubation times

Kawamoto et al. (2013) evaluated the CP degradability values of silages made from soybeans harvested at different growth stages. They reported the rapidly soluble fraction (a) for whole green soybeans (R6) to be 74.4%, while for soybeans that had 50% yellowing pods and leaves (R7), the value for a was determined to be 58.1%. In our study, the a value for GS was found to be 84.19%, which is higher than the values observed for soybean silages. Kawamoto et al. (2013) determined the time dependent degradable protein fractions and the protein degradation rate constants for R6 and R7 to be 20.6%-38.7% and 0.0115 and 0.097, respectively. In our research, the b value was identified to be 12.43%, indicating that guar degrades CP parameters in the rumen more rapidly than soybean, according to results from both studies.

Kawamoto et al. (2013) also reported INSE_CP_ contents for their silage samples from different growth stages, with values of 88.2% for R6 and 83.6% for R7 (k = 0.05). The INSE_CP_ value found for GS in our study was 90.68%, which is comparable to the values reported in their findings. Furthermore, Kawamoto et al. (2013) recorded NDF ratios for R6 and R7 at 40.0% and 41.1%, respectively. These values are higher than those found for guar in our study and may account for the differences observed between soybean silage and GS.

Karayilanli and Ayhan (2017) reported significant differences in the CP degradability values of alfalfa hay harvested at different growth stages (bud formation, beginning of flowering, full flowering, and seed setting). The average CP degradability values for the alfalfa harvested during these stages over a 0–72 h incubation period were determined to be as follows: 37.34% to 87.91%; 35.62% to 88.55%; 32.76% to 81.55%; and 31.61% to 76.31%. In our study, the GH genotype exhibited a CP degradability rate in the rumen of 53.88%–97.02% over the 0–72 h incubation period. These values are higher than the in situ rumen degradability values reported by Karayilanli and Ayhan (2017) for alfalfa.

The researchers noted that with increasing maturity, fresh plants with high moisture content are replaced by those with higher cellulose content, leading to an increase in the DM content of the plants. They reported that the in situ degradability of DM, OM, and CP in alfalfa hay was negatively affected by the increase in structural carbohydrates, specifically cellulose and hemicellulose, during maturation. A strong correlation between in situ degradability and maturity was found: degradability parameters decreased with increasing plant maturity, showing negative correlations with both NDF and ADF contents, but positive correlations with CP and CA contents.In our study, the NDF and ADF ratios were observed to be quite low, and when compared to reported literature, it is considered that these parameters may explain the high degradability values observed. We concluded that the guar plants harvested at 75 days did not develop sufficient cell wall structural components.

## Conclusion

This study evaluated the chemical composition, saponin levels, RFV, fermentation characteristics, and digestibility parameters of silages prepared from dried plant material of the guar plant (*Cyamopsis tetragonoloba*) with various additives. The obtained data were compared with existing literature. The findings indicated that the samples analyzed exhibited particularly high levels of DM and CA compared to many values reported in the literature, while the levels of EE, CF, NDF, ADF, and ADL were lower. Factors such as harvest time, climatic conditions, cultivation techniques, and the use of additives were found to significantly influence the crude protein content.

In terms of RFV, the groups with GH, GS, and GM were categorized within the “best quality” class. Although the addition of molasses and straw slightly reduced the RFV, it maintained the overall quality within acceptable limits. Organic acid analyses revealed that LA levels in the silages met the standards for quality silage, while butyric acid levels remained minimal, effectively preventing undesirable fermentation.

The high saponin levels identified in this study may be attributed to factors such as variety, ecological conditions, maturity stage, and cultivation techniques. Guar bean, under arid conditions, offers high nutritional value and good digestibility (especially in GH, GS, and GM groups), making it a promising alternative summer legume forage. However, considering the relatively high saponin contents which are known to influence palatability and ruminant metabolism and the absence of direct in vivo performance, palatability, or toxicity data in the present study, practical recommendations for widespread use should be made cautiously. Further in vivo feeding trials and assessments of potential anti-nutritional effects are needed before confirming its applicability in ruminant diets.

The results suggest that the guar plant has the potential to serve as a high quality alternative among the limited number of leguminous forage crops that can be produced, especially during the summer. The low NDF and ADF contents offer a significant advantage in terms of digestibility for ruminant feeding. Hence, the guar plant could hold strategic importance for the feed industry if harvest timing, additive ratios, and cultivation conditions are optimized to enhance feed yield and nutritional value.

Future studies are anticipated to provide more comprehensive insights into the feed values of different genotypes, the effects of various additive combinations on fermentation quality, ruminant performance, and economic evaluations. Such research will contribute to a better understanding of the potential of *Cyamopsis tetragonoloba* as roughage.

## Supplementary Information

Below is the link to the electronic supplementary material.


Supplementary Material 1


## Data Availability

The data that support the findings of this study are available from the corresponding author upon reasonable request.
