# Supplementary material for: Guar bean (Cyamopsis tetragonoloba): evaluation as an alternative forage source in ruminants
Source: Trop Anim Health Prod. 2026 Mar 12;58(2):168. doi: 10.1007/s11250-026-04983-1 (PMC12982291; doi:10.1007/s11250-026-04983-1)

**Supplementary Figures**

**Figure S1:** Guar plants at the 75-day maturity stage


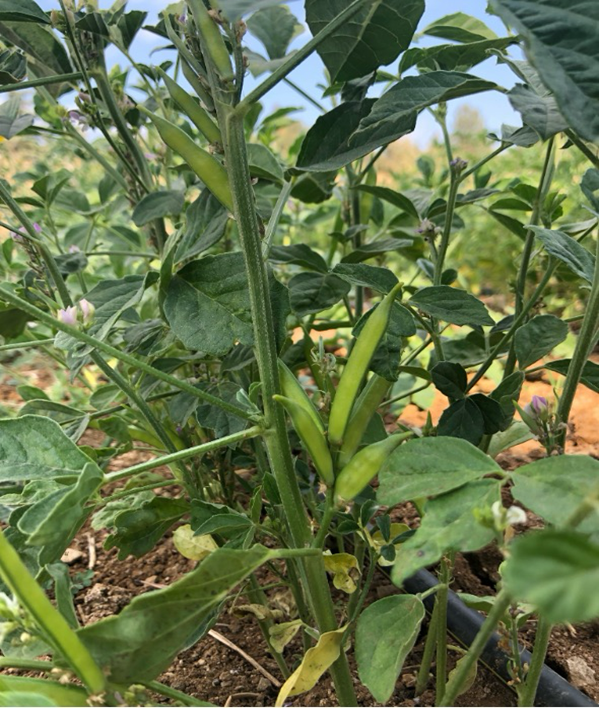


**Figure S2:** Silage Production of Guar Bean: Additive and Non-Additive Groups


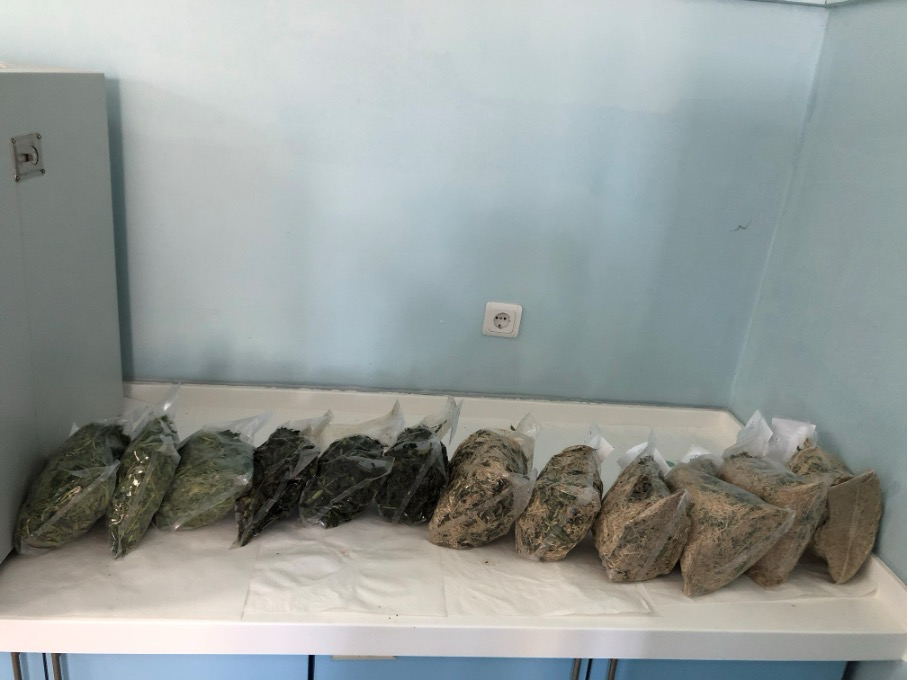

Supplement: Supplementary file 1 — Supplementary Material 1 [file 11250_2026_4983_MOESM1_ESM.docx]
